# Integrative Bioinformatics Analysis for Targeting Hub Genes in Hepatocellular Carcinoma Treatment

**DOI:** 10.2174/0113892029308243240709073945

**Published:** 2024-07-18

**Authors:** Indu Priya Gudivada, Krishna Chaitanya Amajala

**Affiliations:** 1 Department of Biochemistry and Bioinformatics, GITAM School of Science, GITAM (Deemed to be University), Visakhapatnam, 530045, Andhra Pradesh, India

**Keywords:** Liver cancer, gene expression datasets, interaction network, hub genes, drug targets, systems biology, omics studies

## Abstract

**Background:**

The damage in the liver and hepatocytes is where the primary liver cancer begins, and this is referred to as Hepatocellular Carcinoma (HCC). One of the best methods for detecting changes in gene expression of hepatocellular carcinoma is through bioinformatics approaches.

**Objective:**

This study aimed to identify potential drug target(s) hubs mediating HCC progression using computational approaches through gene expression and protein-protein interaction datasets.

**Methodology:**

Four datasets related to HCC were acquired from the GEO database, and Differentially Expressed Genes (DEGs) were identified. Using Evenn, the common genes were chosen. Using the Fun Rich tool, functional associations among the genes were identified. Further, protein-protein interaction networks were predicted using STRING, and hub genes were identified using Cytoscape. The selected hub genes were subjected to GEPIA and Shiny GO analysis for survival analysis and functional enrichment studies for the identified hub genes. The up-regulating genes were further studied for immunohistopathological studies using HPA to identify gene/protein expression in normal *vs* HCC conditions. Drug Bank and Drug Gene Interaction Database were employed to find the reported drug status and targets. Finally, STITCH was performed to identify the functional association between the drugs and the identified hub genes.

**Results:**

The GEO2R analysis for the considered datasets identified 735 upregulating and 284 downregulating DEGs. Functional gene associations were identified through the Fun Rich tool. Further, PPIN network analysis was performed using STRING. A comparative study was carried out between the experimental evidence and the other seven data evidence in STRING, revealing that most proteins in the network were involved in protein-protein interactions. Further, through Cytoscape plugins, the ranking of the genes was analyzed, and densely connected regions were identified, resulting in the selection of the top 20 hub genes involved in HCC pathogenesis. The identified hub genes were: KIF2C, CDK1, TPX2, CEP55, MELK, TTK, BUB1, NCAPG, ASPM, KIF11, CCNA2, HMMR, BUB1B, TOP2A, CENPF, KIF20A, NUSAP1, DLGAP5, PBK, and CCNB2. Further, GEPIA and Shiny GO analyses provided insights into survival ratios and functional enrichment studied for the hub genes. The HPA database studies further found that upregulating genes were involved in changes in protein expression in Normal *vs* HCC tissues. These findings indicated that hub genes were certainly involved in the progression of HCC. STITCH database studies uncovered that existing drug molecules, including sorafenib, regorafenib, cabozantinib, and lenvatinib, could be used as leads to identify novel drugs, and identified hub genes could also be considered as potential and promising drug targets as they are involved in the gene-chemical interaction networks.

**Conclusion:**

The present study involved various integrated bioinformatics approaches, analyzing gene expression and protein-protein interaction datasets, resulting in the identification of 20 top-ranked hubs involved in the progression of HCC. They are KIF2C, CDK1, TPX2, CEP55, MELK, TTK, BUB1, NCAPG, ASPM, KIF11, CCNA2, HMMR, BUB1B, TOP2A, CENPF, KIF20A, NUSAP1, DLGAP5, PBK, and CCNB2. Gene-chemical interaction network studies uncovered that existing drug molecules, including sorafenib, regorafenib, cabozantinib, and lenvatinib, can be used as leads to identify novel drugs, and the identified hub genes can be promising drug targets. The current study underscores the significance of targeting these hub genes and utilizing existing molecules to generate new molecules to combat liver cancer effectively and can be further explored in terms of drug discovery research to develop treatments for HCC.

## 1 INTRODUCTION

The liver is associated with multiple metabolic processes. Its tissues are composed of two histological components: parenchyma and stroma. Hepatocytes comprise the parenchyma cells, and the connective tissues that support the hepatocytes' activity comprise the stromal cells [1, 2]. Hepatocyte death is regarded as liver cancer and is caused by damage to the hepatocytes or hepatic cells [3]. Loss of liver parenchyma cells, which results in inflammation of the liver, is the leading cause of HCC [4]. Around 80% of cases of liver cancer are caused by hepatocellular carcinoma (HCC), which arises in hepatocytes [5]. According to a recent survey, men are the ones most impacted by this HCC. It ranks fifth in terms of the most common cancers diagnosed [6], and it is the second malignant tumour that results in mortality. Aflatoxin exposure, viral hepatitis, or persistent alcohol consumption all contribute to the enormous and abrupt death of hepatocytes [7]. Obesity, diabetes, and hypertriglyceridemia are occasionally additional factors of HCC [8].

Several research studies have been carried out on HCC towards diagnosis, treatment, and prevention, but no proper control method exists. In most cases, liver resection and transplantation are done in the early stages to avoid death [6, 9], as the liver is the only organ that can regrow with the support of various factors, even after removing 70% of the portion [8]. The effect of morbidity and mortality plays a substantial role in postresection and posttransplant survival problems [10]. Therefore, further studies are required to avoid surgery. Identifying novel drug therapies and discovery is required to reduce the carcinogenic effect without damaging other organs.

Bioinformatics is one of the best solutions where high throughput data can be obtained to study and discover novel drug targets and drug candidates for various diseases. In the current study, to understand the carcinogenic effects of HCC, potential drug target(s), hubs mediated through protein-protein interaction networks, and metabolic pathways were identified through the *in silco* approach. Computational methods integrating systems biology approaches with gene expression and protein-protein interaction network datasets offer a holistic understanding of complex biological systems like hepatocellular carcinoma (HCC). Gene expression data illuminates which genes are active, while protein-protein interaction networks reveal how these genes function together. By integrating and analysing these datasets, we can uncover intricate molecular mechanisms driving disease progression, enabling the identification of critical genes and pathways implicated in HCC. This integrated approach is invaluable for deciphering the underlying biology of diseases and facilitating the discovery of novel therapeutic targets and biomarkers [11]. Gene expression datasets of hepatocellular carcinoma were employed to obtain Differentially Expressed Genes (DEGs) collected from GEO2R analysis. Upregulated and downregulated genes were identified by considering the respective logFC values. For the obtained genes, the Protein-Protein Interaction (PPI) network was constructed to identify the hub genes to understand better the molecular mechanisms underlying the onset, development, and management of hepatocellular carcinoma. Cyto-Hubba and Molecular Complex Detection (MCODE) plugins of Cytoscape were used to identify top-ranked genes and densely connected regions, respectively. Gene Expression Profiling Interactive Analysis (GEPIA) performed a survival analysis of top-ranked genes. The ranked genes were further subjected to functional enrichment studies through Shiny GO analysis and Kyoto Encyclopedia of Genes and Genomes (GO and KEGG) databases, revealing the high-level biological system functioning and pathways. Furthermore, the genes were studied for their immunopathological effects virtually using the Human Protein Atlas database (HPA). Finally, using the Drug Bank and Drug Gene Interaction Database, various drugs were studied to identify which drug was suitable for the identified genes, and this was confirmed by the STITCH database.

## 2 MATERIALS AND METHODS

### 2.1 Gene Expression Data Source

Four datasets were selected based on the cause and development of HCC-associated conditions. They were DNA methylation [12], HBV and HCV-associated HCC (viral effect on HCC) [13], Age-related HCC [14], Alcohol-associated HCC [15], and Cirrhosis and tumor in association with HCC [16]. All gene expression datasets were obtained from GEO (Gene Expression Omnibus) (https://www.ncbi.nlm.nih.gov/gds) [17]. The details of the datasets considered for the current study are described in Table **1**.

### 2.2 Processing the Data for DEGs Screening

The GEO2R web application (https://www.ncbi.nlm.nih.gov/geo/geo2r/) [21] was used to identify genes that exhibit differential expression in two or more GEO series samples. For each selected dataset, the groups defined as tumor and non-tumor samples, cirrhotic *vs*. normal, age-specific HCC *vs*. normal, and alcohol *vs*. normal were reanalyzed through ‘GEO2R Analyze’ to identify DEGs. Various parameters like the *p*-value (adjusted p-value <0.05), force normalization, limma precision weight, and the Benjamini Hochberg were verified [22]. The DEGs (upregulating and downregulating genes) were screened based on logFC value ≤2. The DEGs obtained from GEO2R were further analyzed in the EVenn platform (http://www.ehbio.com/test/venn/). It is a versatile tool used to determine genes that are persistently differently expressed across datasets, resulting in the identification of strong, significant biological genes within the intersection of the datasets [23].

### 2.3 Construction and Analysis of Protein-Protein Interaction Networks

Protein-Protein Interaction Network (PPIN) construction and analysis were done using three tools/databases *viz*., Fun Rich Tool, STRING, and Cytoscape. These studies help us to identify the genes that have the highest and strongest interactions mediating HCC.

#### 2.3.1 Identification of Nodes using Fun Rich Tool

Functional enrichment tool (Fun Rich tool) (http://www.funrich.org/) is a software package designed for functional enrichment analysis and visualization of genes and proteins. It is particularly useful for analysing large-scale omics datasets, such as those generated from gene expression or proteomics experiments. This study was used to thoroughly visualize the nodes that have strong interactions and no interaction as well [24]. The genes that were obtained from Evenn were subjected to the Fun Rich tool to identify the non-interacting nodes. This process helps to filter the genes that have no interaction and enables us to study the significant nodes further using the STRING database.

#### 2.3.2 Construction of Protein-Protein Interaction Networks Using STRING Database

The obtained data of interacting nodes were subjected to STRING (Search Tool for Retrieval of Interacting Genes or Proteins) (https://string-db.org/) database, a web tool that forecasts protein-protein interactions [25]. It is used to predict protein function and its relationship with other proteins and to understand the biological processes underlying the molecular level. It assigns confidence scores to each interaction, indicating the reliability of the supporting evidence. The PPIN of all interacting nodes/DEGs of HCC were constructed and ascertained using the STRING database [26]. Usually, the interaction network is represented by seven types of evidence data: text mining, experiments, databases, coexpression, neighborhood, gene fusion, and cooccurrence. We comparatively analyzed the experimental data with all types of data. The upregulating and downregulating gene networks were separately constructed and downloaded to further identify hub genes. The highest number of nodes, edges, and the average node degree were regarded as hub genes.

#### 2.3.3 Visualization and Analysis of Hub Genes Using Cytoscape

Cytoscape is a powerful software platform for visualizing and analysing biological networks (https://cytoscape.org/) [27]. The ranking of the hub nodes in the regulation network was located using CytoHubba. The top overlapping genes and their node degrees were determined by MCC (Maximal Clique Centrality) [28]. Using the MCODE plug-in, densely connected regions in the network were identified with degree cut-off 2, node score cut-off 2, K-core 2, and max depth = 100 [29].

### 2.4 Patient Survival Analysis using GEPIA

A web-based server named GEPIA was used for cancer patient survival (http://gepia2.cancer-pku.cn/#index) [30]. The obtained high-ranked hub genes were subjected to survival analysis against LIHC (Liver Hepatocellular Carcinoma). Overall survival and disease-free survival (or Relapse Free Survival -RFS) were performed for each hub gene, and the results were analyzed through Kaplan Meier Plots. The gene was regarded as statistically significant when the log-p rank level was less than 0.01 [31].

### 2.5 Shiny GO Functional Enrichment Analysis

The shiny GO tool aids in understanding the biological significance of the gene symbols. (http://bioinformatics.sdstate.edu/go/) Functional enrichment studies were carried out on the genes that were identified From Kaplan Meier Plots of GEPIA [32]. It gave information on cellular components, Molecular functions, and biological processes under GO and the KEGG.

### 2.6 Immunohistochemical Staining Evaluation using HPA Database

The Human Protein Atlas (HPA) database (https://www.proteinatlas.org/) was utilized to examine the degree of expression of hub genes, which can be of potential in mediating HCC [33]. This database is intended to research how normal and tumor cells are expressed in liver tissues. The identified hub genes from the Cytoscape were further studied through this database [34].

### 2.7 Reported Drugs and their Gene-chemical Association Studies using Drug-gene Interaction and Drug Bank Databases

The drugs reported for liver cancer treatment were obtained from the literature, and their clinical trial status was identified using the DrugBank database (https://go.drugbank.com/), and Drug-Gene Interaction Database (DGIdb) (https://www.dgidb.org/) was employed to verify whether the identified hub genes are the same drug targets for the reported drugs [35, 36].

### 2.8 Study of Genes Interaction with Chemical Compounds using STITCH Database

STITCH database (http://stitch.embl.de/) [37] was used to find the interaction between proteins and small molecules. Reported drugs were identified from the literature, and 20 hub genes were subjected to the STITCH database to identify the drugs and their interactions.

## 3 RESULTS

### 3.1 Identification of DEGs from the Selected Datasets

**Fig. (1):**
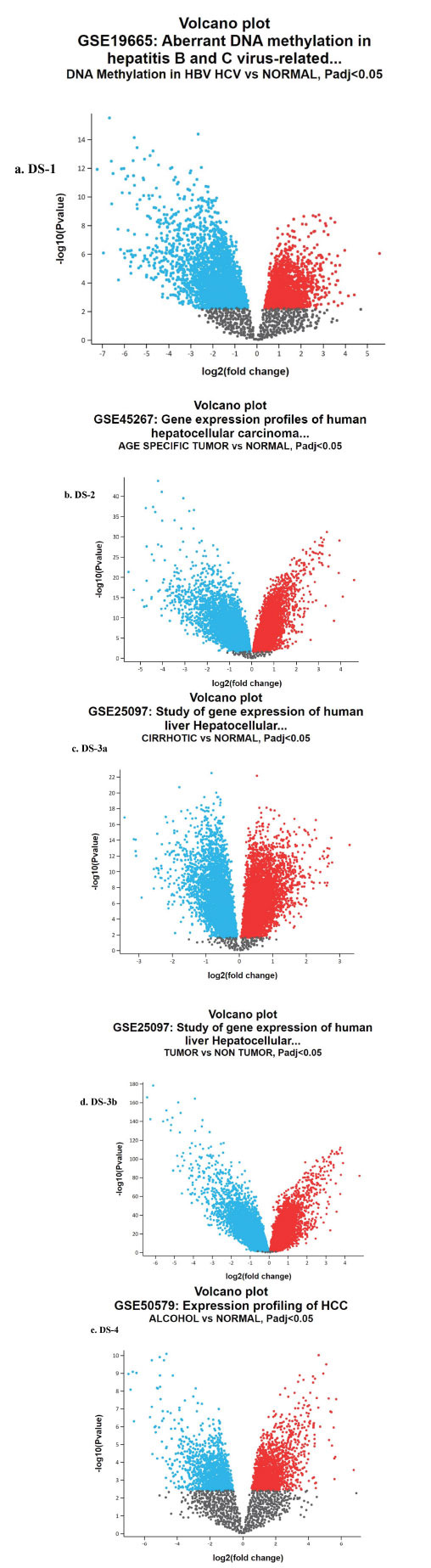
Volcano plots of (**a**) DS-1. DEGs in DNA Methylation in HBV and HCV, (**b**) DS-2 DEGs in Age Specific 35-60, (**c**) DS-3a DEGs in cirrhotic, (**d**) DS-3b DEGs in tumor, (**e**) DS-4 DEGs in Alcohol that are derived from GEO2R analysis. The red and blue colored dots indicate upregulating and downregulating genes, respectively. The black colored dots are the genes that do not show any expression as per the cutoff standard *p*-value of <0.05.

**Fig. (2):**
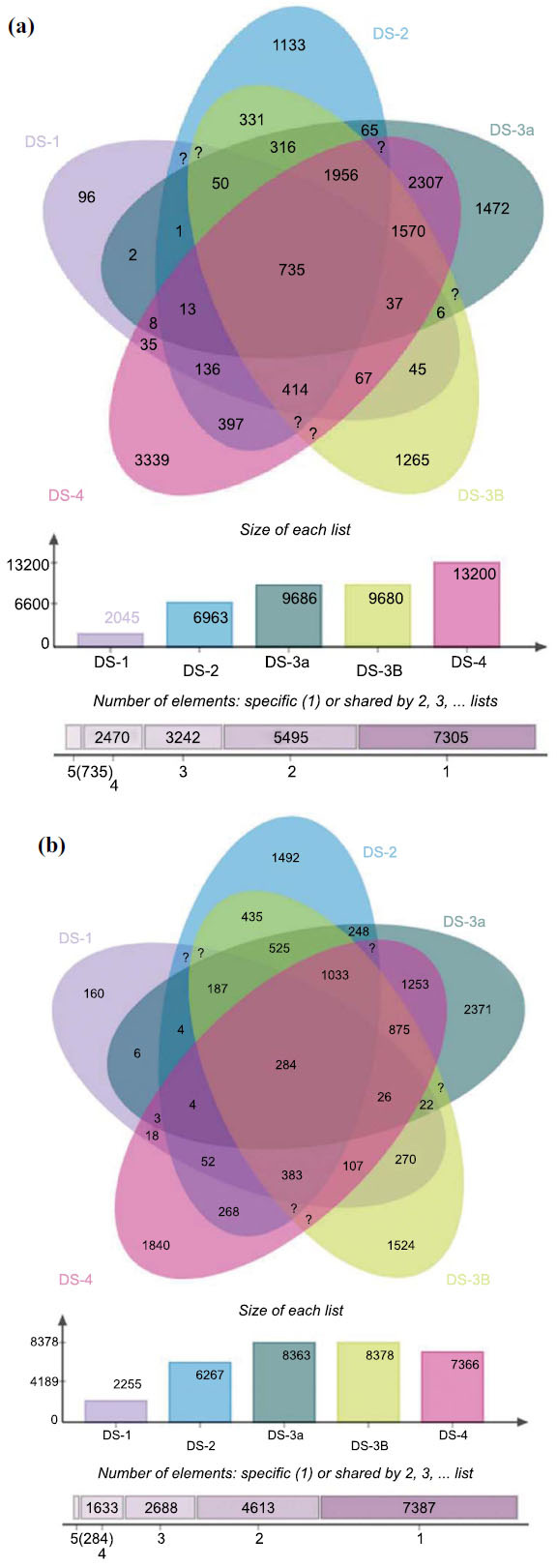
Identification of common genes by intersection analysis from the datasets (**a**) 735 upregulating genes and (**b**) 284 downregulating genes.

From GEO repository, datasets DS-1, DS-2, DS-3a, DS-3b, DS-4 were chosen for the current investigation. A comparative analysis was conducted by creating groups between samples to find genes that were expressed differently. GEO2R analysis resulted in respective volcano plots (Fig. **1**), and significant DEGs with a large fold change (log FC) were selected, *i.e*., the values of 0 to ≤ 2 FC were considered upregulating genes, and values of 0 to ≤-2 FC were considered downregulating genes. The other statistically significant *p*-value was the default of adjusted *p*-value < 0.05 or FDR < 0.05. From the DS-1 volcano plot, 2025 upregulating and 2255 downregulating genes were identified. Similarly, in DS-2 volcano plot, 6964 upregulating, and 6267 downregulating genes; in DS-3 volcano plot, 9688 upregulating, and 8363 down-regulating; in DS-3b volcano plot, 9680 upregulating and 8378 downregulating, and DS-4 volcano plot 13220 up-regulating, and 7366 downregulating genes were identified. A brief overview of the total number of genes identified from each dataset is given in Table **2**. The DEG's intersection of four datasets was performed using the E Venn tool, resulting in identifying common upregulating and downregulating genes in all datasets of HCC (Figs. **2a** and **b**). DS-1, DS-2, DS-3a, b, and DS-4 are represented in violet, blue, green, yellow, and pink color, respectively. The common genes in these datasets (DS) are represented at the center. A total of 735 up-regulating and 284 downregulating genes were identified in common. The list of the 735 upregulating genes and 284 downregulating is provided in Table **S1** of supplementary file.

The question marks are visual indicators of further investigation that might be needed to determine the true status of those elements in the intersection as per the Evenn tool.

### 3.2 PPIN Analysis and Hub Genes Identification

#### 3.2.1 Analysis of Functional Gene Interactions

**Fig. (3):**
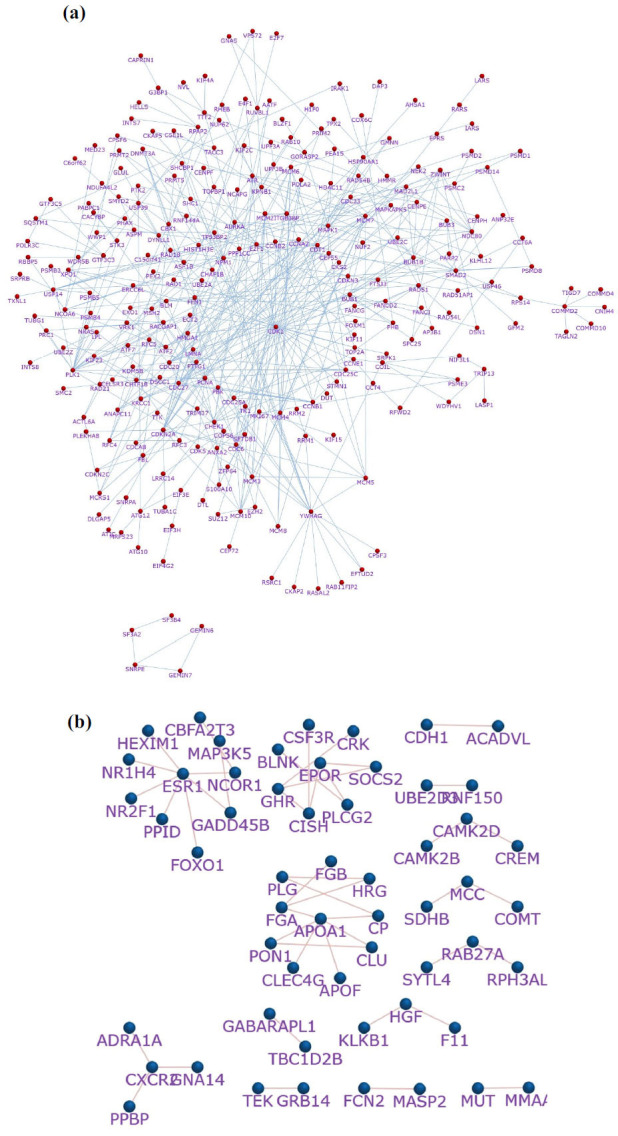
Genes and their interacting partners of (**a**) upregulating genes and (**b**) downregulating genes representing strong interactions.

Using the Funrich tool, the significant gene interactions are visualized. Among 735 upregulating genes, 584 were interacting, and the remaining nodes did not have any interactions. Similarly, in downregulating genes among 284, only 214 genes interacted, whereas other gene nodes did not interact (Figs. **3a** and **b**). The nodes that had no interactions, *i.e*., a single node without any edges in both upregulating and downregulating genes, are presented in Figs. (**S1** and **S2**) of the supplementary file. The genes that had no interactions were removed using various options of Funrich tools, such as the ‘show noninteraction nodes’ option being disabled, and therefore, only strong interactions were selected for further study.

#### 3.2.2 Analysis of PPIN

**Fig. (4):**
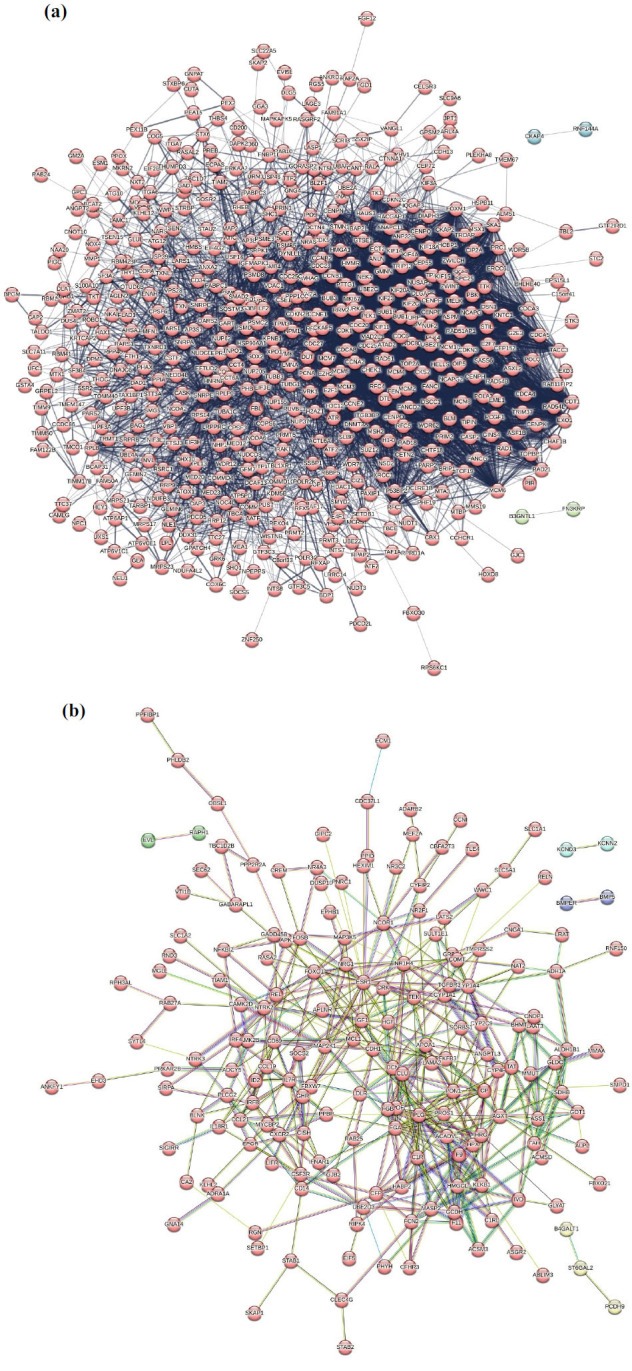
STRING analysis (**a**) PPI network of 583 upregulating genes (**b**) PPI network of 214 downregulating genes. Dark edges indicate strong interactions, light color indicates moderate interactions, and the dotted lines indicate weak interactions between the respective nodes.

After being subjected to Fun Rich analysis, 584 upregulating genes, and 214 downregulating genes were obtained, for which PPINs were constructed and analyzed using the STRING database. This network represents three types of interactions, *i.e*., strong (represented in thick lines) and weak interactions (in thin lines), and dotted lines are computational inferences rather than direct experimental evidence. All seven types of data evidence and experimental evidence data are compared, and network statistics are tabulated in Table **3**. Interestingly, experimental evidence and all evidence data differ with only one node, indicating that the majority of the nodes in the network are involved in protein-protein interactions. The PPI enrichment indicates the biological connection of proteins in the upregulating and downregulation genes in the network. Nodes are represented in three different colors formed after applying the K-means cluster (Figs. **4a** and **b**). The proteins with high correlation and many interactions were analyzed and considered as potential drug target hubs mediating HCC.

#### 3.2.3 Visualization and Identification of Hub Genes

**Fig. (5):**
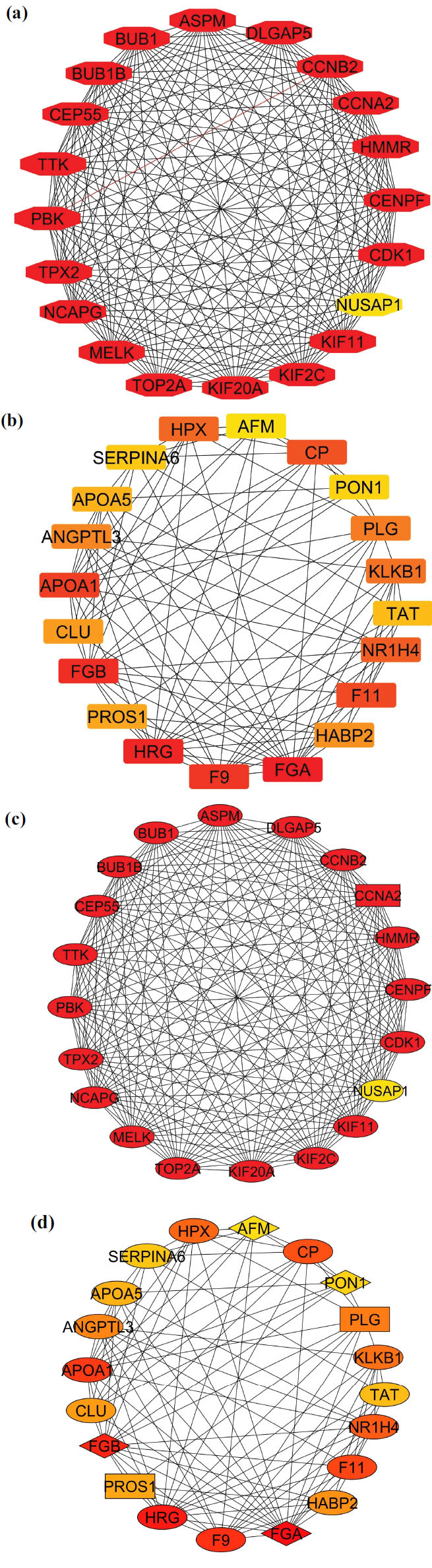
Cytoscape analysis in the selection of top 20 hub genes (**a**) CytoHubba analysis representing the ranking of the nodes, red-colored nodes as one, and the yellow-colored node ranked as 20 in the upregulating network, and (**b**) color varies from red to yellow according to their ranking in the downregulating network. (**c**) MCODE analysis representing CCNA2 as a densely connected region among upregulating and (**d**) FGA and FGB as densely connected regions in the downregulating network.

The obtained nodes and edges of the genes were visualized and further studied using Cytoscape to identify highly interacted hub genes. Using the CytoHubba plugin of Cytoscape, the genes ranking from one to twenty were identified and considered as hub genes (Figs. **5a** and **b**). The identified upregulating hub genes were KIF2C, CDK1, TPX2, CEP55, MELK, TTK, BUB1, NCAPG, ASPM, KIF11, CCNA2, HMMR, BUB1B, TOP2A, CENPF, KIF20A, DLGAP5, PBK, and CCNB2, which were ranked ‘1’ whereas NUSAP1 was ranked ‘20’. The identified downregulating hub genes were FGA (ranked ‘1’), HRG, FGB, F9, APOA1, F11, CP, NR1H4, HPX, KLKB1, PLG, ANGPTL3, HABP2, CLU, PROS1, APOA5, TAT, SERPINA6, PON1, and AFM. Further, the MCODE plugin identified protein clusters in the network based on strong connections. It requires nodes to be connected to others at least twice (node degree and K-core of '2'), a max depth of 100 checks if a seed node interacts within 100 levels, and parameters like “hair cut” and 'Fluff' control cluster density. “Self-looping” is turned off to prevent bias in gene interaction scoring and clustering. After reviewing these parameters, strong connections between the constituent proteins are denoted by a high density of edges in densely linked regions. MCODE results are tabulated in Table **4**. From Figs. (**5c** and **d**), it can be identified that CCNA2 from the up-regulating genes has 19 interactions and is considered densely connected, and in the case of downregulating genes, FGA and FGB have 14 interactions with other genes, PLG has 10, AFM has 8, and PON1 has 7 interactions, respectively.

### 3.3 GEPIA-survival Analysis

The findings of the GEPIA analysis on 20 hub genes for overall survival and disease-free survival in LIHC (Liver Hepatocellular Carcinoma) are shown in the Kaplan Meier graph represented in Figs. (**6a** and **b**) and tabulated in Table **5**. The X-axis represents the time or duration of treated genes, and the Y-axis represents the probability of surviving. The Hazard ratio (HR) is an average value given by comparing HR in the treated group with HR in the control group. The overall survival of HCC patients varies with changes in the genes. Similarly, disease-free survival was also performed, and the genes with log-rank *p*-value <0.01 were considered statistically significant. The results state that among 20 hub-upregulating genes, 19 were identified as core genes that can be considered potential molecular biomarkers for HCC. The genes KIF2C, KIF11, BUB1B, NUSAP1, KIF20A, CEP55, CDK1, TOP2A, CCNA2, HMMR, DLGAP5, ASPM, TPX2, NCAPG, CENPF, BUB1, MELK, TTK, and CCNB2 had high mortality and shortest survival rate. The Disease-Free Survival was performed on these genes, and it was identified that all 20 genes of upregulating showed <0.01. Similarly, this study was carried out on the top 20 down-regulating genes; the details are mentioned in Table **6** and the respective Kaplan Meier plots are provided in the supplementary file (Figs. **S3a** and **b**).

### 3.4 Shiny GO Analysis

**Fig. (7):**
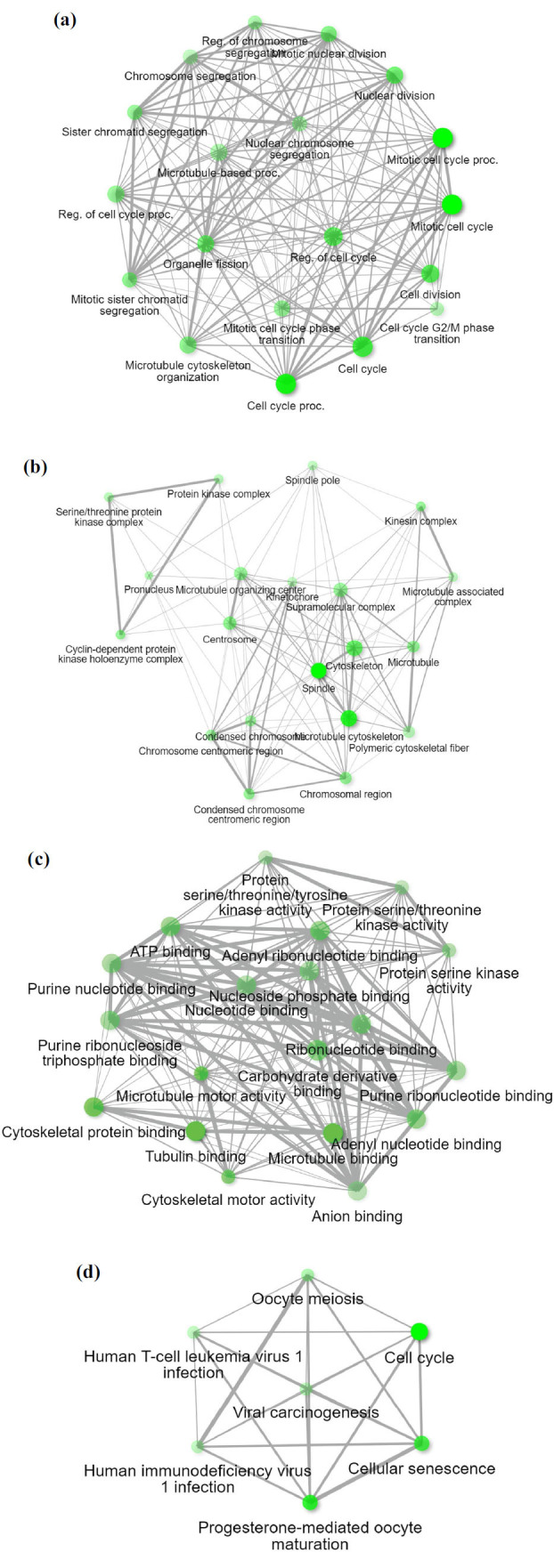
Network structures from Shiny GO analysis for Function enrichment analysis for upregulating genes, representing (**a**) Interconnection of biological processes, (**b**) Interconnection of cellular components, (**c**) Interconnection of molecular functions, and (**d**) KEGG Pathway Annotation. The bright, fluorescent green circle represents activities highly expressed by the genes.

Shiny GO enrichment analyses were performed to explore the GO and KEGG functional annotation for the genes with p rank <0.01 from GEPIA studies. The Gene Ontology study was significantly enriched with a) cell cycle process, b) mitotic cell division, c) nuclear division, d) cell division, e) organelle fission, f) microtubule cytoskeleton, g) spindle, h) supramolecular complex, i) ATP binding, j) Adenyl ribonucleotide, and k) nucleotide binding, *etc*., which were observed in upregulating and similarly in downregulating a) response to wound healing, b) blood, hemostasis, c) external encapsulating structure, d) regulation of body fluids, e) collagen-containing extracellular matrix, and f) signaling receptor binding complement. KEGG annotation for the upregulating genes revealed the following pathways: a) cell cycle, b) oocyte meiosis, c) progesterone-mediated oocyte maturation, d) Viral carcinogenesis, e) P53 signaling pathway, and f) cellular senescence. The pathways and cellular function enrichment data are tabulated and represented in Table **7** and (Fig. **7**), respectively. All data related to downregulating genes are provided in the supplementary file (Fig. **S4**).

### 3.5 Immunohistopathological Studies on Gene’s Protein Level in Normal Tissue and HCC

**Fig. (8):**
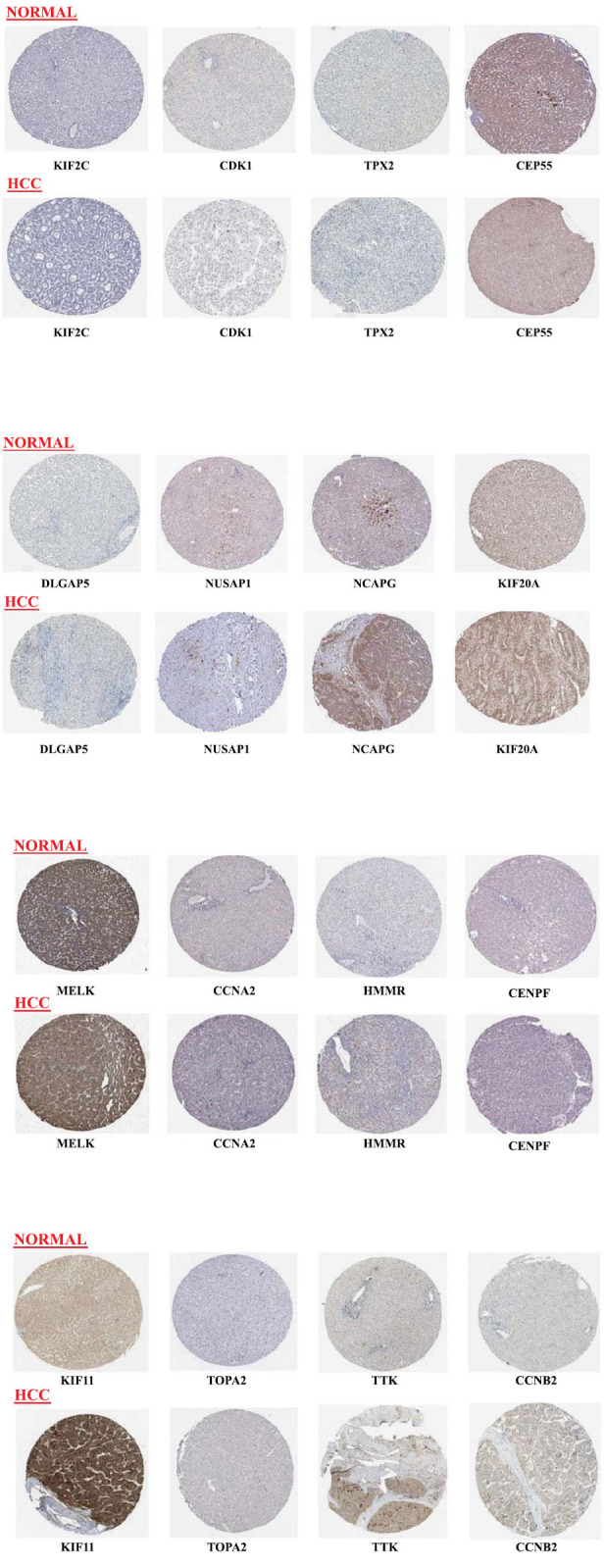
Immunohistopathological studies of upregulating genes from the HPA database. Normal and HCC tissue images were compared for visual confirmation, abundance, and localization of gene expression patterns, enabling the identification of upregulating gene changes in protein expression. The expression of KIF2C, CDK1, TPX2, CEP55, DLGAP5, NUSAP1, NCAPG, KIF20A, MELK, CCNA2, HMMR, CENPF, KIF11, TOPA2, TTK, and CCNB2 were significantly upregulating in HCC tissues.

The difference in the protein level was exhibited using the HAP database. KIF2C, CDK1, TPX2, CEP55, DLGAP5, NUSAP1, NCAPG, KIF20A, MELK, CCNA2, HMMR, CENPF, KIF11, TOPA2, TTK, and CCNB2 are the genes taken for immunohistopathology studies. They are described as negatively stained in healthy tissues and positively stained in HCC tissues. This demonstrates that the expression of these genes was substantially higher in tissues with HCC when compared to healthy tissues (Fig. **8**).

### 3.6 Reported Drugs and their Clinical Status

The drugs for the treatment of HCC were collected from the literature, and their activity and clinical trial status were studied using the drug bank database. The names of the drugs were Atezolizumab, Bevacizumab, Cabozantinib, Ramucirumab, Durvalumab, Futibatinib, Tremelimumab, Infitratinib, Ipilimumab, Pembrolizumab, Lenvatinib, Sorafenib, Nivolumab, Pemigatinid, and Regorafenib, which are presented in Table **8**. These drugs are given in combination with chemotherapy. Furthermore, DGIdb was used to observe whether these drugs target our study's hub genes. It was found that the 20 hub genes identified in the current investigation were not directly involved in targeting by the reported drugs.

### 3.7 Identification of Drugs Targeting Hub Genes

**Fig. (9):**
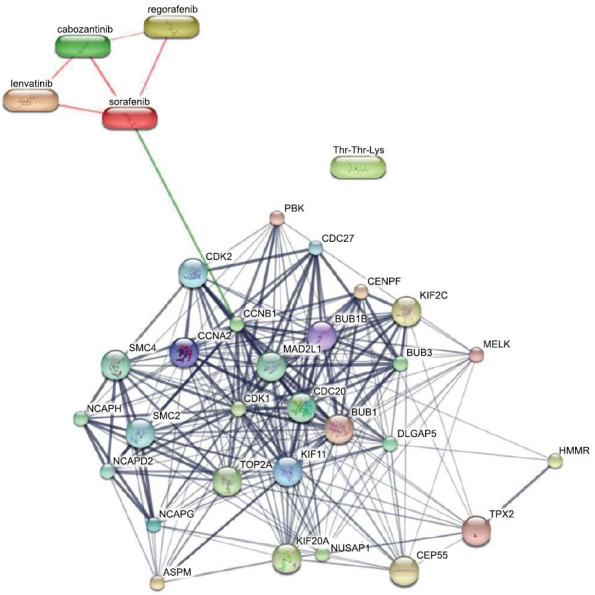
Drug-hub gene interaction and association through STITCH database. The red line indicates a direct drug-drug interaction effective in combinational therapy. The green line indicates strong predicted interaction and relation with the gene CCNB1, the thick black line indicates a strong interaction between the genes, and the thin line indicates weak interactions among the genes.

The drugs reported, and the hub genes of this present study were subjected to the STITCH database. It was identified that four drugs, cabozantinib, regorafenib, lenvatinib, and sorafenib, had high confidence interaction between them, indicating a reliable relationship between the molecules. Furthermore, the drug sorafenib was found to have a strong predicted interaction with the CCNB1 gene in connection with other identified hub genes (Fig. **9**). CCNB1 was found to be exhibiting stronger or higher confidence in interaction and relation with the identified hub genes CDK1, CCNA2, BUB1, CENPF, BUB1B, and KIF11. Other genes like NUSAP1, TPX2, CEP55, KIF20, MELK, HMMR, KIF2C, ASPM, NCAPG, and KIF20A exhibited lower confidence of interactions in the gene-chemical interaction network. The comprehensive analysis of gene-chemical interaction networks suggests that all identified hub genes have potential as drug targets. Additionally, the molecules already known to interact with these genes could serve as promising starting points for developing therapies against hepatocellular carcinoma.

## 4 DISCUSSION

Presently, various studies have been carried out in the field of drug discovery, including the identification of biomarkers associated with molecular biology and genomics. These approaches often help to increase the current therapies and approaches for treating various cancers, including HCC. However, the mortality rate of HCC is increasing. Finding the correct cause by which it occurs and making an early diagnosis can help the patient overcome the problem. Therefore, there is a need to determine the underlying reason for hepatocyte destruction, which is the disease's earliest stage. The most common form of liver cancer is hepatocellular carcinoma. Bioinformatics approaches can be used to examine data to get a greater comprehension of the molecular processes underlying the onset of cancer and the identification of potential therapeutic, diagnostic, and drug discovery targets [52]. Using HCC-specific gene expression datasets and protein interaction network studies, 20 upregulating and 20 downregulating genes were identified, respectively. These genes were further analyzed for overall survival and disease-free analysis using log-p rank <0.01 as the cutoff criterion. AFM [53], HRG [54], PON1 [55], CLU [56], TAT [57], and APOA5 [58], were the genes identified as significant genes in downregulating. On further analysis of these genes through Shiny GO, they were enriched in response to wound healing, blood hemostasis, external encapsulating structure, regulation of body fluids, collagen-containing extracellular matrix, signaling receptor binding complement. All these genes can be considered for various other studies related to gene therapy or immunotherapy.

The 20 upregulating hub genes named KIF2C, KIF11, BUB1B, NUSAP1, KIF20A, CEP55, CDK1, TOP2A, CCNA2, HMMR, DLGAP5, ASPM, TPX2, NCAPG, CENPF, BUB1, MELK, TTK, and CCNB2 were further studied based on the log p rank (considering less than 0.01 as the cut of criterion). According to Shiny GO analysis, these genes were enriched for activities such as ATP binding, Adenyl ribonucleotide, and nucleotide binding, division of the nucleus, splitting of cells, organelle fission, microtubule cytoskeleton, spindle, and supramolecular complex.

During the cell cycle, the member of the Kinesin family, KIF2C, was found in the cytoplasm [59]. It plays a role in kinetochore-microtubule connection, spindle assembly, chromosomal assembly, and segregation, all of which are crucial for mitosis. Increasing the amount of KIF2C protein in the microtubule made chromosomal instability worse [60, 61]. A recent study revealed that KIF2C regulates the kinetics of double-strand DNA breaks, improving the rate of DNA damage repair and preserving genomic stability [62]. Wnt/ βcatenin increases KIF2C gene expression in hepatocellular cancer. KIF2C expression may enhance mTORC1 signaling transmission, which in turn may facilitate the growth, invasion, and motility of cells in cancer [63]. CDK1 is the primary control of the cell cycle. The group of CDKs that have endured through evolution includes CDK1 [64]. It influences every stage of cell division, including cytokinesis, nuclear breakdown, chromosomal compression and segregation, the G1/S phase transition, and entrance to the developmental cycle of the cell quiescence, and even drives mitosis and S phases in the absence of CDK2 [65]. Targeting CDK1 during the period of G2/M transition halts cell cycle progression in HCC cells. Theoretically, inhibition of CDK1 overexpression increases hepatoma cell senescence and death, whereas excessive expression of CDK1 in HCC is associated with abnormal cell cycle activity [66]. TPX2 is necessary for the production of microtubules and controls cell mobility throughout important biological activities, such as cell proliferation, division, and apoptosis. HCC cells can develop resistance to the four Tyrosine Kinase Inhibitors (TKIs) as well as four cytotoxic chemotherapeutic medicines when exposed to TPX2 [67].

By supporting both symmetric and asymmetric neurogenic divisions, the ASPM gene plays a crucial part in the cycle-regulating differentiation of brain progenitor cells. Additionally, ASPM, which undergoes positive selection throughout the evolutionary process of the genetic basis of brain growth, is essential for the appropriate movement of neurons during carcinogenesis [68]. Recent studies on ASPM are involved in the positive regulation of the Wnt/- catenin transmission of the signal system and in the fact that overexpressing -catenin can reverse the faulty neurogenesis brought on by mice lacking sufficient ASPM. The adult brain also expresses ASPM [69]. In HCC, Wnt signaling activation in malignant tumour increases cancer progression, while Wnt signaling stimulates cancer stem cells and improves stemness by boosting Wnt-Dvl-3-catenin signaling [70]. KIF11, a member of the kinase family, is crucial to many biological activities, such as mitosis and the transport of vesicles and organelles within cells [71]. The premature division of sister chromatids and the uneven chromosomal distribution that arises due to the upregulation of KIF11 proteins during mitosis may further contribute to progeny cell aneuploidy [72]. KIF11 is expressed more frequently in a variety of cancers and is associated with a bad prognosis for cancer, according to recent studies [73]. The genomic instability brought on by KIF11 abnormalities promotes the spread of cancer, for instance, by accelerating invasion and metastasis [72]. By mediating the Wnt/-catenin signaling pathway's activity, ASPM and KIF11 accelerate the malignant development of HCC [74].

The advancement and reappearance of carcinoma of the liver, carcinoma of the prostate, pancreatic ductal adenocarcinoma, and multiple additional malignancies have been linked to overexpression of BUB1B [75]. During lung adenocarcinoma metastasis, BUB1B may regulate anchorage-independent proliferation and survival, which will aid in the tumor's dissemination. Furthermore, significant chromosomal damage and apoptosis were seen in human cancer cells when the BUB1B level was lowered or BUB1B kinase activity was inhibited [76]. Since KIF20A is overexpressed in the cell types that proliferate and is especially linked to the mitotic state of dividing cells, stem/progenitor cells from a variety of tissues are expected to include it [77]. Patients with bladder cancer who have high tumor-evaluated stages that are advanced and have poor outcomes are linked to high KIF20A expression. KIF20A's prognostic importance has also been assessed in different solid tumors, where its critical function in tumour metastasis and cell proliferation has been established [78]. A significant factor in chromosomal instability and carcinogenesis is abnormal TOP2A expression, strongly correlated with the initiation, incursion, course of treatment, and prognosis of malignant tumours; demonstrated to be an effective therapy against cancer [79]. As a new oncogenic gene, CCNA2 controls the growth and death of cancer cells. CCNA2 may increase resistance to chemotherapy, cancer metastases, relapse, and aggressive behavior in cancer [80]. It is necessary for both embryonic cells and the blood-forming lineage, and it performs crucial functions in controlling the cell cycle during the G1/S and G2/M phases [81].

Among various tumors, in liver, stomach, lung, and bladder cancer, HMMR regulates proliferation and metastasis, preserves stemness, and confers resistance to treatment [82]. Elevated HMMR was strongly linked to a worse prognosis in a more advanced pathologic stage. Upregulation of HMMR markedly inhibited or expedited the processes of invasion, cell proliferation, cell cycle transit, and migration. In terms of mechanics, HMMR may interact with AURKA and increase the amount of AURKA protein by blocking this process, which in turn causes the mTOR/AKT axis to become activated [83]. Numerous malignancies, such as including cancer of the prostate, hepatic carcinoma (HCC), and other carcinomas, have been discovered to express CENPF aberrantly [84]. The cell cycle controls CENPF expression, which gradually rises during the cycle, peaks in the G2/M phase, and then declines once mitosis is complete. In addition to dramatically reducing EMT cell division and colony formation, suppressing CENPF expression also increased sensitivity to anoikis-induced apoptosis and global phosphorylation [85]. MELK is involved in several different procedures, like the division of cells and the cell cycle, the death of cells, the processing of RNA, and the development of the embryo [86]. MELK participates in several protein interactions that impact various stages of carcinogenesis. It is a cell-cycle modulator that is necessary for the division of mitosis. The relationship between MELK and immune-related checkpoint expression, respectively, immune cell infiltration, and immune cell markers in HCC [87]. The overall survival rate of patients with gastric cancer was positively correlated with TTK expression, and a high level of TTK overexpression was linked to an increased risk of cancer recurrence [88]. TTK is required for chromosomal orientation of the centromere during centrosome replication and mitosis. It also takes part in the division of cells and proliferation. TTK knockdown promoted apoptosis, reduced proliferation, and prevented Akt-mTOR communication from being activated [89]. Several cancers, including bladder, breast, and lung cancers, showed aberrant expression of CCNB2. The poor prognosis of individuals suffering from HCC was caused by its upregulation [90]. Through CDK activation, CCNB2 is involved in the G2/M phase transition of the eukaryotic cell cycle. Similarly, TOP2A, CCNB2 expression is distributed and is dispersed inside a cell cluster, providing a module indicator of cell proliferation inside a cell cluster [91]. Defects in CCNB2 led to the failure of the G2/M checkpoint during the cell cycle, which in turn brought on gene alterations and the development of cancer [92].

BUB1 plays a crucial part in setting up a mitotic spindle checkpoint and chromosomal alignment [93]. Meanwhile, inhibition of BUB1 expression prevented the development of cancerous liver cell lines, and overexpression of BUB1 markedly accelerated cell growth [94]. NUSAP1 governs the cell cycle by encouraging microtubule aggregation, which is essential for spindle assembly and creation [95]. Periodically throughout the cell cycle, NUSAP1 protein expression fluctuates, increasing during interkinesis and reducing following mitosis [96, 97]. Prior studies revealed that a variety of tumors, including liver cancer, have aberrantly high levels of NUSAP1 [98]. DNA methyltransferase's mRNA expression was markedly reduced when NUSAP1 was silenced, but not the expression of an oncogene linked to gliomas. By decreasing apoptosis and accelerating cell cycle progression, NuSAP1 aided in the growth of liver cancer. The aberrant level of NUSAP1 expression may contribute to the spread of liver cancer [99]. Hepatoma upregulating protein (HURP), also known as DLGAP5, is a protein that regulates the cycle of cells [100]. The diversified DLGAP5 protein has been the subject of numerous research studies, and functional relationships have been revealed between it and the development of liver cancer tumors [101]. It participates in a variety of biological functions that occur inside cells, such as the phase of the cell, spindle development, microtubule organization, and motor activity [102]. CCP55 is known to be necessary for abscission, the last stage of cytokinesis, and has also been discovered to be elevated in some cancers [103]. Recent information on CEP55's role in the regulation of the PI3K/AKT pathway, midbody fate, and stem cells raises the possibility that CEP55 may have a broader function that promotes development and long-term survival on a number of different levels. Therefore, the finding that CEP55 promotes tumour survival and growth by being overexpressed in numerous malignancies is not unexpected [104]. The PI3K/AKT pathway is upregulated when CEP55 is overexpressed, which encourages the invasion and motility of cells in hepatocellular carcinoma (HCC) and lung adenocarcinoma (LAC) [105]. SMC-free condensing during meiosis and mitosis, a complex component called a complex subunit, is in charge of preserving and condensing the chromosome [106]. NCAPG shows increased migration and proliferation in HCC since it has high expression in HCC castration-resistant cancers of the prostate and melanoma [107]. Through the PI3K/AKT signaling system, NCAPG aids in the growth, migration, and inhibition of death of cells in HCC. When NCAPG was elevated or inhibited *in vitro* and *in vivo*, the PI3K/AKT/FOXO4 pathway was improperly energized, and the gene expression of proteins linked to apoptosis was changed [108].

There are many drugs that are successful in treating patients at various stages of HCC. Some of them have been approved by the FDA, and a few are in various phases of clinical trials. They are Atezolizumab, Bevacizumab, Cabozantinib, Ramucirumab, Durvalumab, Futibatinib, Tremelimumab, Infitratinib, Ipilimumab, Pembrolizumab, Lenvatinib, Sorafenib, Nivolumab, Pemigatinid, and Regorafenib respectively. Atezolizumab and Bevacizumab are given in combination, which is involved in anti-Egfr therapy. This is an immune checkpoint inhibitor, treated both before and after resection of the liver, especially in patients where HCC cannot be removed completely [51]. Sorafenib is used to block the VEGF receptor, which blocks the growth of blood vessels. Other drugs, such as Ramucirumab, Futibatinib, Infitratinib, Ipilimumab, Pembrolizumab, Lenvatinib Nivolumab, Pemigatinid, and Regorafenib are given to patients who were treated with sorafenib sometimes in combination or individually depending on patient’s condition. This is based on elevated levels of the tumor marker AFP(Alfa-fetoprotein) [109]. Tremelimumab and Durvalumab were given to patients after resection.

Among these drugs, the direct targets of top hub genes (*i.e*., KIF2C, KIF11, BUB1B, NUSAP1, KIF20A, CEP55, CDK1, TOP2A, CCNA2, HMMR, DLGAP5, ASPM, TPX2, NCAPG, CENPF, BUB1, MELK, TTK, CCNB2) were analysed using Stitch Database. It was identified that four drugs, *viz*., sorafenib, regorafenib, cabozantinib, and lenvatinib, could be target drugs for these genes, which are associated with CCNB1. With the comparison of previous studies, we determined that the 10 hub genes that were identified in the current study participate actively in the development of HCC. Anyhow, the underlying mechanisms to control the disease need more research to confirm the results. This research may have some limitations due to the lack of experimental evidence. Further robust data analysis, gene coexpression analysis, and RNA analysis will help to find the expression difference of these genes. However, the combined bioinformatics analysis is more accurate in identifying the possibilities of diagnosing HCC at the early stages. Drug discovery research to develop treatments uses these genes as a platform. However, to determine their characteristics and represent them as potential molecular indicators in the therapy of HCC, more studies are required.

Prior to these studies, several bioinformatics approaches were conducted to identify hub genes in HCC. However, the research we conducted still has a few clear benefits: Firstly, the dataset that we have selected was based on the criterion of various conditions of HCC formation. They included DNA methylation, HBV and HCV, age, specific cirrhosis, and alcohol. As a result, the discovered genes have special guiding relevance for prompt diagnosis and effective therapy. Secondly, we considered all rank:1 genes that were obtained from CytoHubba Plugin. Our research may contribute to a better understanding of the molecular underpinnings of HCC development and establish a framework for the advancement of HCC diagnosis and therapy. Thirdly, high expression of these genes has a remarkable impact on the overall survival caused by overexpression of viral infections and alcohol methylation, and these genes could be used as prognostic biomarkers for HCC. Finally, we used the HPA database to verify the tissue expression and studied the drugs that are reported for HCC, using drug bank and DGIdb, and found that existing drugs have association and interaction with hub gene network through CCNB1 which is a related gene to CCNB2 hub gene.

## CONCLUSION

Through systematic analysis of gene expression data related to hepatocellular carcinoma (HCC), 735 upregulating and 284 downregulating DEGs were identified using the Evenn tool. By leveraging bioinformatics tools, such as Fun Rich Tool, 584 upregulating, and 214 downregulating genes were found to have functional associations that were further investigated through the STRING database. A comparative study was carried out between the experimental evidence and the other seven data evidence in STRING, revealing that most proteins in the network were involved in protein-protein interactions. Further, through Cytoscape plugins, the ranking of the genes was analyzed, and densely connected regions were identified, resulting in the selection of the top 20 hub genes involved in HCC pathogenesis. The identified hub genes included: KIF2C, CDK1, TPX2, CEP55, MELK, TTK, BUB1, NCAPG, ASPM, KIF11, CCNA2, HMMR, BUB1B, TOP2A, CENPF, KIF20A, NUSAP1, DLGAP5, PBK, and CCNB2. Further, GEPIA and Shiny GO analyses provided insights into survival ratios and functional enrichment studied for the hub genes. The HPA database studies further found that upregulating genes were involved in changes in protein expression in Normal *vs* HCC tissues. These findings indicate that hub genes are certainly involved in the progression of HCC. Gene-chemical network interaction studies found that drugs like sorafenib, regorafenib, cabozantinib, and lenvatinib could lead to the discovery of new medications. Also, hub genes identified in the gene-chemical interaction networks show promise as potential drug targets. The current approach and findings can initiate new possibilities for understanding novel targets to diagnose and treat HCC early. These findings also shed light on the molecular mechanisms underlying HCC and present potential avenues for targeted drug development.

## AUTHORS’ CONTRIBUTIONS

The authors confirm contribution to the paper as follows: study conception and design: K.C. Amajala; draft manuscript: I.P. Gudivada. All authors reviewed the results and approved the final version of the manuscript.

## Figures and Tables

**Fig. (6a, b):**
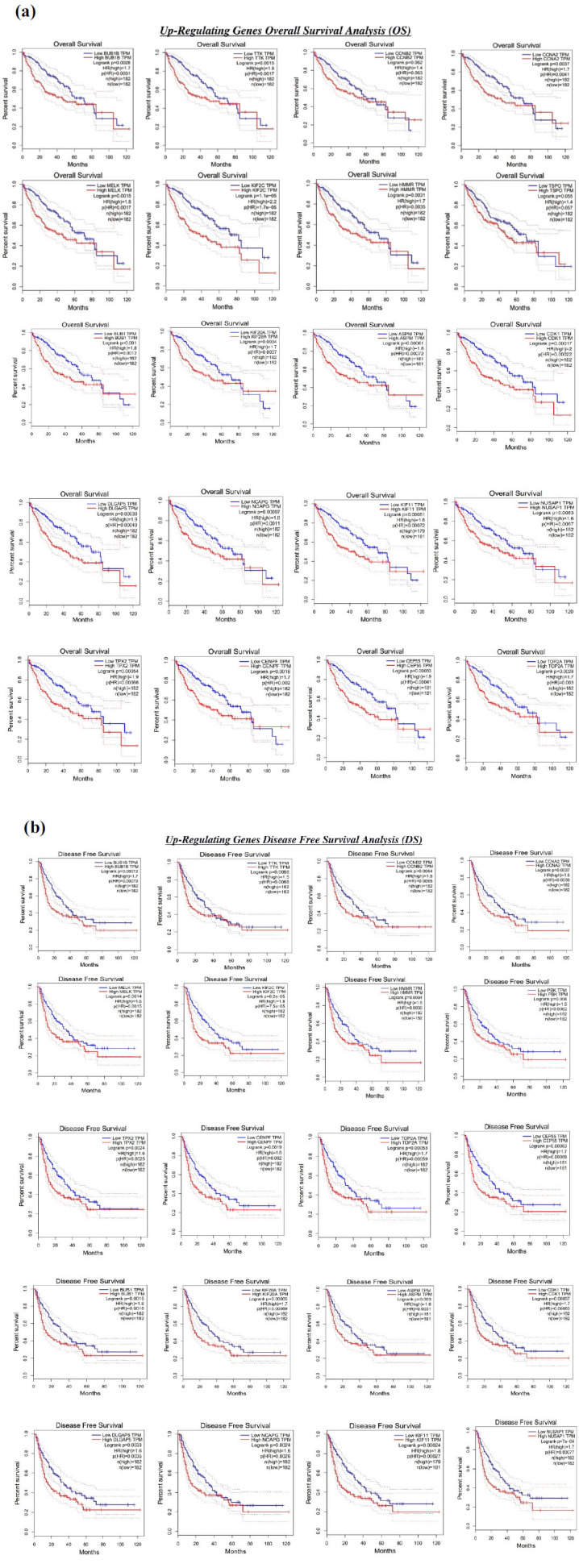
Overall Survival and Disease-free survival of upregulating genes obtained from Kaplan Meier plot using GEPIA. In the forest plot, each point represents hazard ratio (HR), and the points on either side represent 95% confidence intervals. The proportion of survival to month is shown in each graph. Time is plotted on the X-axis, and survival chance is on the Y-axis. Genes that are upregulated and have changed in expression are shown by a red line, whereas a blue line shows genes that have not changed. A statistically significant p-rank of <0.01 is considered.

**Table 1:** GEO datasets related to HCC.

**S. No.**	**Datasets**	**Source**	**Define Groups**	**References**
Dataset-1 (DS-1)	GSE19665	DNA methylation in HBV and HCV [GPL570]	Normal *Vs* Methylation	[13]
Dataset-2 (DS-2)	GSE45267	Human hepatocellular gene expression profile [GPL570]	Age Specific 35-60 *Vs* Normal	[18]
Dataset-3 (DS-3)	GSE25097	Gene expression in HCC [GPL60876]	a. Cirrhosis *Vs* Healthy b. Tumor *Vs* Non-Tumor	[19]
Dataset-4 (DS-4)	GSE50579	Expression profiling of HCC [14550]	Alcohol *Vs* Normal	[20]

**Table 2:** Number of upregulating and downregulating DEGs in each dataset.

**Dataset**		**Condition Specific HCC Dataset**	**Total Genes**	**Upregulating**	**Downregulating**
DS-1		DNA methylation	4280	2025	2255
DS-2		Age specific	13231	6964	6267
DS-3	a	Cirrhosis	18051	9688	8363
	b	Tumor	18058	9680	8378
DS-4		Alcohol	20586	13220	7366

**Table 3:** PPI network data of upregulating and downregulating genes.

**Network Status**	**Upregulating**		**Downregulating**	
	**All Evidences**	**Experimental Evidence**	**All Evidences**	**Experimental Evidence**
No. of nodes	584	583	213	212
No. of edges	1043	1016	485	26
Average node degree	4.31	3.49	4.55	0.245
Average clustering coefficient	0.401	0.404	0.397	0.139
Expected no. of edges	337	690	252	11
PPI enrichment *p*-value	<1.0e^-16^	<1.0e^-16^	<1.0e^-16^	4.17e^-05^

**Table 4:** MCODE results for both upregulating and downregulating genes.

**Parameters**	**Upregulating**	**Downregulating**
**Network scoring:** Including loops	False	False
Degree cutoff	2	2
**Cluster findings:** Node score cutoff	0.2	0.2
Haircut	True	True
Fluff	False	False
K score	2	2
Max. depth from seed	100	100
Cluster	1	1
Score	20	10.316
Nodes	20	20
Edges	190	98
Node IDs	BUB1, KIF11, CCNB2, BUB1B, CEP55, TPX2, PBK, NUSAP1, HMMR, NCAPG, CDK1, TOP2A, DLGAP5, KIF2C, CENPF, KIF20A, ASPM, MELK, TTK, CCNA2	CP, APOA5, ANGPTL3, CLU, F11, FGB, SERPINA6, NR1H4, PLG, PON1, TAT, HRG, FGA, F9, PROS1, KLKB1, HPX, AFM, HABP2, APOA1 PLG, TAT, ANGPTL3, F11, KLKB1,

**Table 5:** List of upregulating genes based on log P rank and hazard ratio data from Kaplan Meier plot.

**S. No.**	**Genes**	**Overall Survival**		**S. No.**	**Genes**	**Disease-free Survival**	
		**Log-P Rank**	**Hazard Ratio**			**Log-P Rank**	**Hazard Ratio**
1.	KIF2C	1.10E-05	2.2	1.	KIF2C	6.2e-0.5	1.8
2.	CDK1	0.00017	2	2.	KIF11	0.00024	1.8
3.	TPX2	0.00054	1.9	3.	BUB1B	0.00072	1.7
4.	CEP55	0.00033	1.9	4.	NUSAP1	7.00E-04	1.7
5.	MELK	0.0017	1.8	5.	KIF20A	0.00065	1.7
6.	TTK	0.0015	1.8	6.	CEP55	0.00063	1.7
7.	BUB1	0.001	1.8	7.	CDK1	0.00057	1.7
8.	NCAPG	0.00097	1.8	8.	TOP2A	0.00053	1.7
9.	ASPM	0.00061	1.8	9.	CCNA2	0.0037	1.6
10.	KIF11	0.00061	1.8	10.	HMMR	0.0034	1.6
11.	CCNA2	0.0037	1.7	11.	DLGAP5	0.0033	1.6
12.	HMMR	0.0031	1.7	12.	ASPM	0.003	1.6
13.	BUB1B	0.0028	1.7	13.	TPX2	0.0024	1.6
14.	TOP2A	0.0028	1.7	14.	NCAPG	0.0024	1.6
15.	CENPF	0.0018	1.7	15.	CENPF	0.0019	1.6
16.	KIF20A	0.0034	1.6	16.	BUB1	0.0015	1.6
17.	NUSAP1	0.00063	1.6	17.	MELK	0.0014	1.6
18.	DLGAP5	0.00033	1.6	18.	TTK	0.0088	1.5
19.	PBK	0.055	1.4	19.	CCNB2	0.0064	1.5
20.	CCNB2	0.052	1.4	20.	PBK	0.006	1.5

**Table 6:** List of downregulating genes based on log P rank and hazard ratio data from Kaplan Meier plot.

**S. No.**	**Genes**	**Overall Survival**		**S. No.**	**Genes**	**Disease-free Survival**	
		**Log-P Rank**	**Hazard Ratio**			**Log-P Rank**	**Hazard Ratio**
1.	AFM	0.0013	0.56	1.	HRG	0.00037	0.064
2.	PON1	0.0017	0.57	2.	FGB	0.0011	0.6
3.	HPX	0.0043	0.6	3.	CLU	0.007	0.66
4.	TAT	0.0067	0.62	4.	APOA5	0.0084	0.67
5.	HRG	0.0083	0.63	5.	TAT	0.011	0.68
6.	FGA	0.017	0.65	6.	FGA	0.013	0.68
7.	SERPINA6	0.025	0.67	7.	PLG	0.022	0.71
8.	FGB	0.029	0.068	8.	AFM	0.025	0.71
9.	F9	0.034	0.59	9.	HPX	0.041	0.73
10.	CLU	0.034	0.69	10.	PON1	0.047	0.74
11.	PLG	0.076	0.73	11.	F9	0.12	0.68
12.	F11	0.086	0.74	12.	SERPINA6	0.14	0.8
13.	KLKB1	0.11	0.75	13.	ANGPTL3	0.21	0.82
14.	ANGPTL3	0.17	0.78	14.	F11	0.43	0.89
15.	APOA1	0.18	0.79	15.	HABP2	0.45	0.89
16.	PROS1	0.18	0.79	16.	CP	0.57	1.1
17.	APOA5	0.26	0.82	17.	NR1H4	0.57	0.92
18.	CP	0.37	1.2	18.	PROS1	0.58	1.1
19.	HABP2	0.41	0.86	19.	APOA1	0.64	0.93
20.	NR1H4	0.88	0.97	20.	KLKB1	0.66	0.94

**Table 7:** List of up and down-regulated genes with their gene ontologies and KEGG annotations.

**Gene Ontology**				
-	**Up Regulating Genes**	**Activity**	**Down-Regulating Genes**	**Activity**
**Biological Process**	KIF2C, KIF11, BUB1B, NUSAP1, KIF20A, CEP55, CDK1, TOP2A, CCNA2, HMMR, ASPM, DLGAP5, TPX2, NCAPG CENPF, BUB1, MELK, TTK, CCNB2	Cell cycle process, Mitotic cell division, Nuclear division, Cell division, Organelle fission	AFM, PON1, HPX, TAT, HGR, FGB	Wound healing, blood, hemostasis, external encapsulating structure
**Cellular Components**		Microtubule cytoskeleton, Spindle, Supramolecular complex		Regulation of body fluids, collagen containing extracellular matrix
**Molecular Function**		ATP binding, Adenyl ribonucleotide Nucleotide binding		Signaling receptor binding complement
**KEGG Pathway**				
**Pathways**	BUB1, BUB1B, CCNA2, CCNB2, CDK1, TTK,	Cell cycle, Oocyte meiosis, Progesterone mediated oocyte maturation P53 signaling pathway Cellular senescence Viral carcinogenesis	TAT, FGB	Ubiquinone and other terpenoid-quinone biosynthesis coagulation cascade PPAR signaling pathway

**Table 8:** Summary of drugs that are reported, activity, and status of clinical trials.

**S.NO.**	**Drugs Reported in HCC**	**Combination**	**Activity**	**Status**	**Trail Phase**	**References**
1.	Atezolizumab	Bevacizumab	Metastatic HCC and patients who were is treated for the first time	Recruiting	4	[36]
2.	Bevacizumab	---	---	Recruiting	4	[38]
3.	Cabozantinib	Sorafenib	Hepatocyte growth factor	Completed	4	[39]
4.	Ramucirumab	Bevacizumab Oxaliplatin	Prevents VEGF stimulation and downregulation of proliferation, permeability, and migration	Completed	3	[39, 40]
5.	Durvalumab	Tremelimumab	Programmed cell death	Recruiting	3	[41]
6.	Futibatinib	---	Inhibits fibroblastin growth factor receptor 2 Treated in patients who were under treatment. Downstream cancer pathways	Recruiting	2	[42]
7.	Tremelimumab	Durvalumab	Decreased tumor growth	Active not Recruiting	3	[43]
8.	Infitratinib	---	Fibroblast growth factor 1 inhibitor	-	-	[44]
9.	Ipilimumab	Nivolumab	Metastatic and unrespectable myeloma on patients treated with sorafenib	Active not Recruiting	3	[45, 46]
10.	Pembrolizumab	Monotherapy	In Patients who treated with Sorafenib Programmed cell death	Active not Recruiting	3	[46]
11.	Lenvatinib	Firstline treatment	Inhibits the kinase activity	Completed	4	[47]
12.	Sorafenib	Monotherapy	Kinase inhibitor	Completed	4	[48]
13.	Nivolumab	Relatlimab	Programmed cell death. Treated in patients > 12 years	Active not Recruiting	3	[49]
14.	Pemigatinid	---	Fibroblastin growth receptors	-	-	[50]
15.	Regorafenib	Sorafenib	Kinase inhibitor	Completed	3	[51]

## Data Availability

The data related to the current study are available from the corresponding author and will be provided on a reasonable request.

## LIST OF ABBREVIATIONS

AFM: Afamin
ANGPTL3: Angiopoietin-like 3
APOA1: Apolipoprotein A-1
APOA5: Apolipoprotein A-5
ASPM: Abnormal Spindle Like Microcephaly- Associated
BP: Biological Process
BUB1: Budding Uninhibited Benzimidazoles-1
BUB1B: Mitotic Checkpoint Serene and Threonine Kinase B
CC: Cellular Components
CCNA2: Cyclin A2
CCNB2: Cyclin B2
CDK1: Cyclin Dependent Kinase
CENPF: Centromere Protein F
CLU: Clusterin
CP: Connective Polypeptide -1
DEGs: Differentially Expressed Genes
DFS: Disease Free Survival
DLGAP5: Disks Large- Associated Protein 5
DS: Dataset
F11: Coagulation Factor 11
F9: Coagulation Factor 9
FGA: Fibrinogen Alfa Chain
FGB: Fibrinogen Beta Rich
GEPIA: Gene Expression Profiling Interactive Analysis
GO: Gene Ontology
HABP2: Hyaluronan Binding Protein 2
HMMR: Hyaluronan Mediated Motility Receptor
HPA: Human Protein Atlas
HPX: Hemopexin
HR: Hazard Ratio
HRG: Histidine Rich Glycoprotein
KEGG: Kyoto Encyclopedia Genes and Genomic
KIF11: Kinase Family Member 11
KIF20A: Kinase Family Member 20A
KIF2C: Kinase Family Member 2C
KLKB1: Kallikrein B1
MCODE: Molecular Complex Detection
MELK: Maternal Embryonic Leucine Zipper Kinase
MF: Molecular Function
NCAPG: Non -SMC Condensing I Complex Subunit G
NCBI: National Center for Biotechnology Information
NR1H4: Nuclear Receptor Subfamily 1Group H Member 4
NUSAP1: Nucleolar and Spindle Associated Protein 1
OS: Overall Survival
PBK: PDZ Binding Kinase
PLG: Plasminogen
PON1: Paraoxonase
PPI: Protein -Protein Interaction
PROS1: Plasma protein S-1
SERPINA6: Serpin Family A Member 6
STRING: Search Tool for the Retrieval of Interacting Genes
TAT: Trans- Activator of Transcription
TOP2A: Topoisomerase 2 Alpha
TPX2: TPX2 Microtubule Nucleation Factor
TTK: Threonine Tyrosine Kinase
