# Supplementary material for: Integrative Bioinformatics Analysis for Targeting Hub Genes in Hepatocellular Carcinoma Treatment
Source: Curr Genomics. 2024 Jul 18;26(1):48–80. doi: 10.2174/0113892029308243240709073945 (PMC11793067; doi:10.2174/0113892029308243240709073945)
Supplement: Supplementary file 1 [file CG-26-1-48_SD1.pdf]

Supplementary Material

Integrative Bioinformatics Analysis for Targeting Hub Genes in Hepatocellular Carcinoma Treatment

Indu Priya Gudivada[1] and Krishna Chaitanya Amajala[1,*]

[1]*Department of Biochemistry and Bioinformatics, GITAM School of Science, GITAM (Deemed to be University), Visakhapatnam, 530045, Andhra Pradesh, India*

Table S1. List of up and down regulating genes.

**List of (DEGs)**
**Up-regulating (735)**
AATF, ABCF2, ABHD12, ACTL6A, AGBL5, AHSA1, AIFM2, ALG6, ALMS1, ANGPT2, ANKRD27, ANLN, ANO10, ANP32E, ANXA2, AP3B1, AP3S1, ARL4A, ASAP2, ASF1B, ASPM, ASTN2, ASXL2, ATAD2, ATF2, ATIC, ATOX1, ATP5H, ATP6AP1, ATR, AURKA, AVL9, B3GNTL1, B4GALT3, BAG2, BCAP31, BCAT2, BDP1, BLM, BLZF1, BOD1, BOLA3, BPGM, BRIP1,BUB1, BUB1B, BUB3, C9orf40, CACYBP, CAMLG, CANT1, CAP2, CAPN10, CAPRIN1, CASK, CASP2, CBX1, CCDC34, CCHCR1, CCNA2, CCNB1, CCNB2, CCNE1, CCNE2, CCT4, CCT6A, CD34, CDC20, CDC25A, CDC25C, CDC27, CDC6, CDCA2, CDH13, CDK1, CDK5, CDKN2A, CDKN2B, CDKN2C, CDKN3, CDT1, CELSR3, CENPE, CENPF, CENPH, CENPI, CENPK, CENPL, CENPM, CENPN, CEP55, CETN2, CHAF1B, CHEK1, CHTF18, CIZ1, CKAP2, CKAP2L, CKAP4, CKAP5, CKS2, CNIH4, CNOT10, CNPY2, COG2, COIL, COL15A1, COMMD2, COPA, COPS6, COX6C, CPSF3, CPSF6, CRNDE, CSE1L, CSTF2, CTNNA1, CUTA, CXorf36, CYP2R1, DAD1, DAP3, DAPK2, DARS2, DCLRE1B, DCTN4, DDX31, DEPDC1B, DHX30, DIAPH3, DLAT, DLG5, DLGAP5, DNAJC6, DNMT3A, DPH2, DPM3, DSCC1, DSN1, DTL, DTYMK, DUS4L, DUT, DYM, DYNLL1, E2F1, E2F7, E2F8, E4F1, ECT2, EFCAB2, EFNA4, EFTUD2, EIF2B4, EIF3E, EIF3H, EIF4G2, ELOVL5, EME1, ENAH, EPRS, ESM1, EVI5L, EXD3, EXO1, EXOG, EZH2, FAM50A, FAM91A1, FANCD2, FANCG, FANCI, FBL, FBXO30, FBXO38, FBXO43, FEN1, FGD1, FGF12, FIGNL1, FKTN, FLAD1, FLVCR1, FN3KRP, FNBP1L, FOXM1, FSD1L, FTH1, FTSJ3, G2E3, G3BP1, GAD1, GART, GAS2L3, GEMIN6, GFM2, GGA3, GINS4, GJC1, GLA, GLUL, GM2A, GMNN, GMPS, GNAS, GNG4, GNPAT, GOLPH3L, GOLT1B, GORASP2, GOSR2, GPATCH4, GPC3,GPR107, GPR137C, GPR19, GPSM2, GRK6, GRPEL2, GSTA4, GTF2IRD1, GTF3C3, GTF3C5, GTPBP2, GTSE1, H1F0, H2AFZ, HAUS3, HAX1, HDAC11, HELLS, HES6, HEY1, HHAT, HIST1H3E, HJURP, HMBS, HMGA1, HMMR, HN1, HNRNPU, HOXD8, HRCT1, HSPB11, IAR, IFT81, IGF2BP2, IGSF3, ILF2, IMMP1L, INTS2, INTS7, INTS8, IPO9, IQGAP3, IRAK1, ITGA6, ITGA7, ITGB3BP, JMJD4, KDM5B, KIF11, KIF14, KIF15, KIF18A, KIF18B, KIF20A, KIF23, KIF2C, KIF3A, KIF4A, KLHL12, KNTC1, KPNB1, KRTCAP2, LAGE3, LAMC1, LANCL1, LARS, LASP1, LMBR1, LMNA, LPL, LRP11,LRPPRC, LRRC14,LYSMD1, MAD2L1, MANBAL, MAP2, MAPK1, MAPKAPK5, MCM10, MCM2, MCRS1, MDK, MEA1, MELK, MESP1, METTL3, MEX3C, MFAP3, MFN1, MKI67, MKRN2, MMP1, MMS19, MND1, MRPL13, MSH2, MSX1, MTA3, MTBP, MTX1, NAA20, NARS, NCAPG, NCAPG2, NDRG3, NDUFA4L2, NDUFB3, NEDD4L, NEIL3, NEU1, NHP2, NIF3L1, NKAP, NKIRAS2, NLE1, NOC4L, NOL7, NOX4, NPC1, NPEPPS, NPM1, NR2C2AP, NRAS, NSMCE2, NT5M, NUDCD1, NUDT1, NUF2, NUSAP1, NVL, NXT2, OIP5, OSBP2, OTUB2, OTUD6B, PABPC1, PARP2, PARS2, PAXIP1, PBK, PCGF1, PCNA, PDCD2L, PDCD5, PEA15, PEX11B, PEX2, PHAX, PHB, PHF19, PHOSPHO2, PIGC, PIR, PLEKHA8, PLK1, PLK4, PLOD3, PLVAP, POGK, POLA2, POLQ, POLR2K, POLR3C, PPIA, PPOX, PPP1CC, PRC1, PREB, PRIM2, PRKAA2, PRMT2, PRR11, PSMB3, PSMC2, PSMD1, PSMD2, PSME3, PTGFRN, PTK2, PTPRG, PTTG1, PUS7, RAB10, RAB11FIP2, RAB24, RABIF, RACGAP1, RAD1, RAD21, RAD51AP1, RAD54B, RAD54L, RALA, RAP2A, RARS, RASAL2, RASGRF2, RBBP5, RBM12B, RCCD1, REXO4, RFC3, RFWD2, RFX5, RFXAP, RGS5, RHEB, RIOK2, RNF144A, ROBO1, RPAP2, RPL8, RPLP0, RPRD1A, RPS14, RPS6KC1, RRM1, RRM2, RRP12, RRP9, RSRC1, RUFY1, RUVBL1, SAE1, SASS6, SCFD2, SCRIB, SEC22C, SESTD1, SF3A2, SF3B4, SHC1, SHCBP1, SHQ1, SKA1, SKA3, SKAP2, SLBP, SLC7A11, SLC9A6, SMA2, SMC2, SMG5, SMOX, SMYD2, SNORA74A, SNRPA, SNRPC,SNRPE, SOCS5, SOX2, SPATA5L1, SPATS2, SPCS1, SPIN3, SQSTM1, SRPK1, SRPRB, SRXN1, SSBP1, SSR2, SSX2IP, STAU2,

STC1, STC2, STIL, STK3, STMN1, STRA13, STRBP, STT3A, STX6, STXBP6, SUZ12, TACC3, TAF1A,T AGLN2, TAL-DO1, TARBP1, TAX1BP1, TBC1D7, TBCA, TBCE, TBL1XR1, TBL2, TCF19, TDRKH, THBS4, THOC2, THY1, TAM2, TIGD1, TIGD7, TIMM9, TIPIN, TK1, TKT, TLCD1, TMCO1, TMEM106C, TMUB2, TNPO1, TOMM40 ,TOP2A, TOPBP1, TP53BP2, TPM3, TPX2, TRIP13, TRMT1, TRMT12, TROAP, TROVE2, TSEN15, TSEN2, TTC5, TTF2, TTK, TTL, TUBA1C, TUBB3, TUBG1, TUFT1, TULP3, TWISTNB, TXN, TXNL1, TXNRD1, UBAP2, UBAP2L, UBE2A, UBL4A, UFC1, ULK4, UPF3A, UPF3B, URM1, UTP15, UXS1, VANGL1, VASH2, VBP1, VDAC1, VPS28, VRK1, VSIG10, WDHD1, WDR12, WDR5B, WDYHV1, WHSC1, WWP1, XPO1, XPR1, XRCC1, YIF1B, YIPF3, YWHAG, ZBTB9, ZC3H3, ZCCHC4, ZCRB1, ZFP62, ZFYVE26, ZKSCAN5, ZMAT2, ZNF2, ZNF746, ZRANB3, ZSWIM1, ZWILCH, ZWINT
**Down-regulating (284)**
ABLIM3, ACAA1, ACADVL, ACMSD, ACOT12, ACSM3, ADAMTSL3, ADARB2, ADCY5, ADH1A, ADH4, ADRA1A, AFM, AGPAT2, AGXT, AGXT2, AKIRIN1, AKNA, ALDH1B1, ALPL, AMDHD1,ANGPTL3,ANKFY1,ANTXR2,ANXA10,APLNR,APOA1,APOA5,ASGR2,ASS1,ATOH8,B4GALT1, BHMT, BLNK, C1R, C1RL, CA2, CAMK2B, CAMK2D, CBFA2T3, CBR4, CCDC25, CCDC69, CCNI, CD14, CDC37L1, CFP, CCL19, CXCL14, CDH1, CFHR3, CHRNA10, CHST7, CISH, CLN8, CLRN3, CLU, CMTM6, CNGA1, COL6A6, COMT, CP, CPT2, CREM, CRK, CSF3R, CXCR2, CYB5D2, CYFIP2, CYP2C9, CYP2J2, CYP3A4, CYP4F2, CYP4V2, CYS1, DAPK1, DCUN1D3, DDB2, DEPDC7, DNAJC22, DUSP10, EIF5, EPHB1, EPOR, ERLIN1, ESR1, ETFDH, EVL, F11, F9, FAH, FBXO21, FBXW7, FCAR, FGA, FGB, FNIP2, FOLH1B, FOSB, FOXO1, FREM2, FRMD4B, GABARAPL1, GADD45B, GCDH, GIPC2, GJB2, GLDC, GLOD5, GLT1D1, GLYAT, GOT1, GPR180, GRB14, HABP2, HAO1, HECA, HEXIM1, HMGCL, HMGCS2, HOOK1, HPX, HRG, HSD17B2, ID2, IFNAR1, IGF1, IL18R1, INTS6, IRF4, IRF8, IVD, KBTBD11, KCND3, KCNK17, KCNK5, KDM6B, KLHL2, KLKB1, KYNU, LATS2, LDLR, LIFR, LNX2, LPIN2, LRAT, LRRN3, LYRM1, MAN1C1, MAP2K1, MAP3K5, MASP2, MBNL2, MCC, MCL1, MEF2A, MGLL, MMAA, MPDU1, MSRA, MUT, MYCBP2, N4BP2L1, NAAA, NAT2, NCOR1, NDEL1, NDRG2, NFKBIZ, NR1H4, NR2F1, NR3C2, NR4A3, NRG1, NSUN6, NTRK2, NTRK3, OBSL1, PALM2, PCDH9, PDE8A, PDLIM5, PFKFB3, PHLDB2, PHYH, PID1, PLA2G16, PLCG2, PLG, PSCR4, PNRC1, PON1, PPBP, PPFIBP1, PPID, PPM1K, PPP1R3B, PPP2R2A, PRDX4, PRG2, PROS1, PROSC, PRR5, PRRG4, RAB27A, RANBP3L, RAPH1, RASA2, RASGEF1B, RCAN1, RCL1, REL, RFX2, RGN, RIPK4, RND3, RNF150, RPAIN, RPH3AL, SAT2, SDHB, SEC62, SEMA5A, SERPINA6, SETBP1, SH3TC1, SIGIRR, SIRPA, SKAP1, SLC10A1, SLC1A1, SLC7A2, SMPD1, SOCS2, SORBS1, SOX6, SPRYD4, SRD5A1, ST6GAL2, ST6GALNAC6, STAB1, STARD5, SYTL4, TAT, TBC1D2B, TBX15, TCTEX1D1, TGFBR3, TIAM1, TLE4, TMPRSS2, TPST1, TRAM2, TRIM15, TSPAN9, TUBE1, UBE2D3, UBL3, UGP2, VTI1B, WDR86, WWC1, YPEL2, ZCCHC24, ZFP1, ZGPAT, DCN, KCNN2, MASP2, FCN2, EHD3, TEK, PRKAR2B, GYS2, RND3, IGF1, FOSB, SPP2, ABCA9, RELN, RAB25, LAMA2, ADH4, GBA3, APOF, CYP1A1, IL7R, SLC1A2, CYP39A1, CCL21, BMP5, STAB2, BCO2, CCDC3, ECM1, BMPER, HGF, CLEC4G, GNA14, SPIC, SCIMPHSD17B13, CCBE1, LPA, CYP3A4, RBMS3, SULT1E1, OIT3, SULT1B1, CD69, CNDP1, C8ORF4, SLC5A1, GHR,

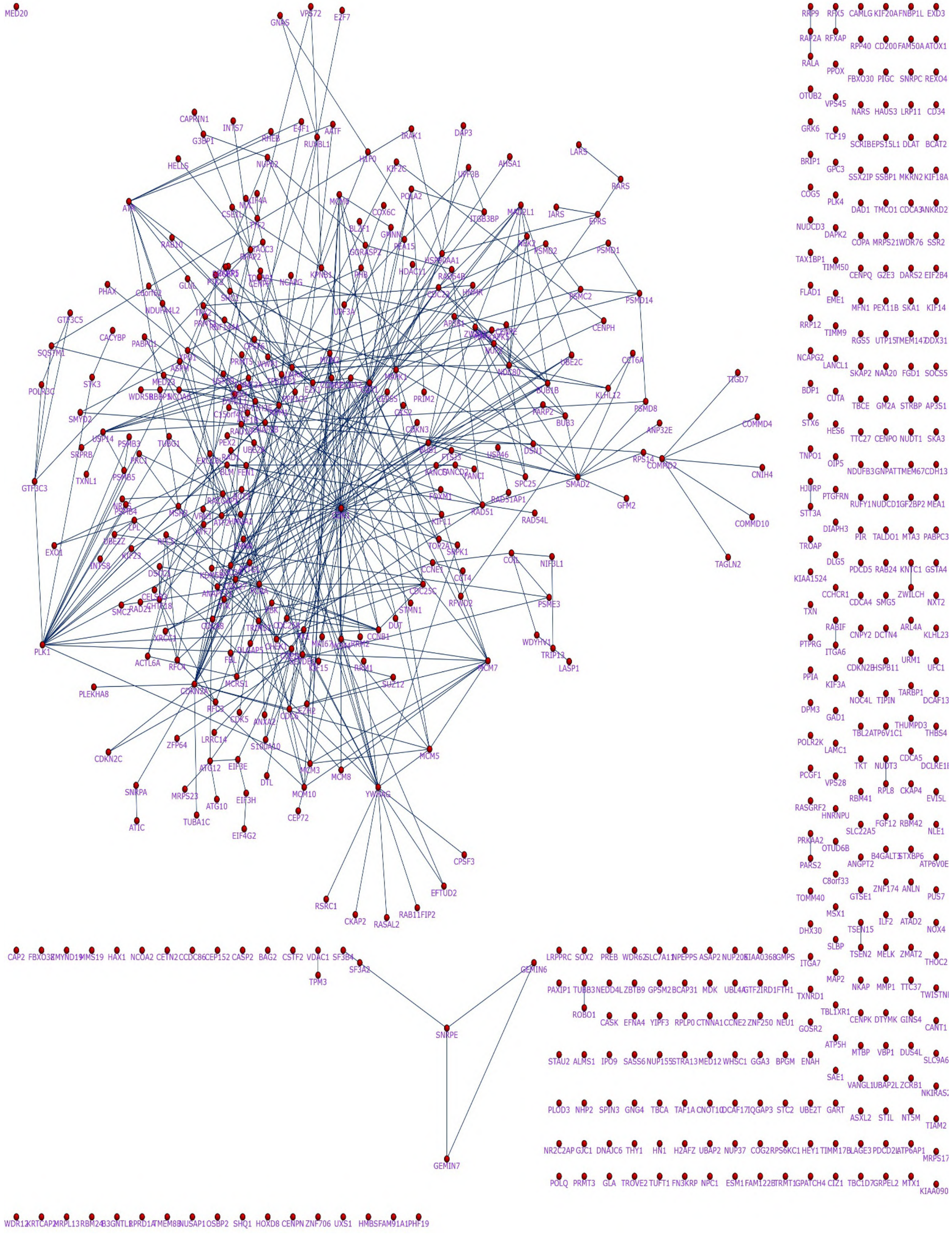

Fig. (S1). Red-colored nodes represent the upregulating genes, blue-colored edges represent interactions, and The nodes with no interactions are represented as single nodes without any edges.

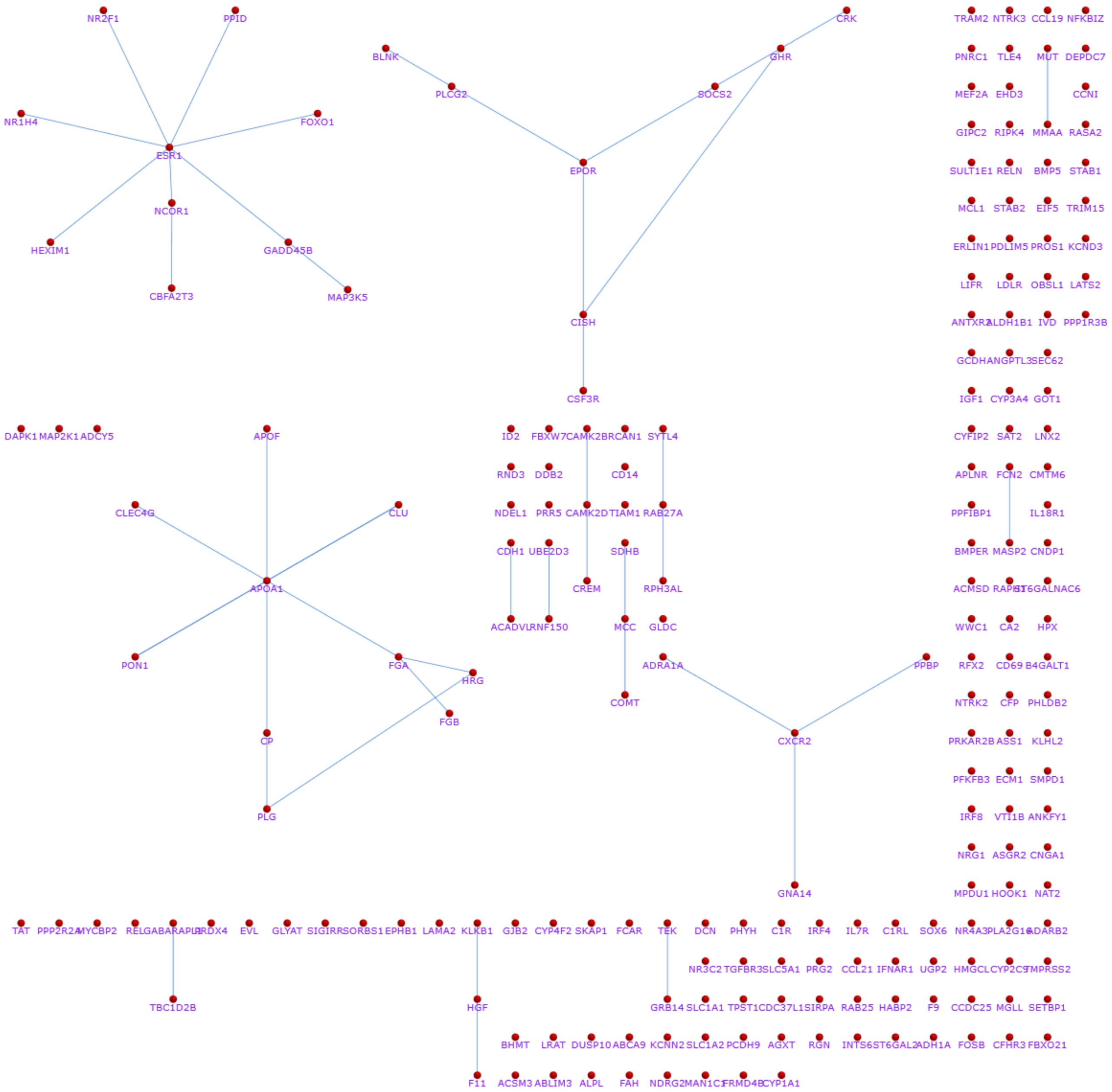

Fig (S2). Red-colored nodes represent the downregulating genes, blue-colored edges represent interactions, and The nodes with no interactions are represented as single nodes without any edges.

GEPIA-Survival Analysis

The 20 downregulating genes viz. ANGPTL3, CP, F11, PROS1, CLU, FGB, PLG, SERPINA6, HABP2, NR1H4, TAT, KLKB1, F9, HPX, HRG, APOA1, APOA5, FGA, AFM, PON1 are further studied through GEPIA database. (Fig. **S3 a, b**). Shiny GO enrichment analysis examines the GO and KEGG for the genes with p rank <0.01. (Fig. **S4**).

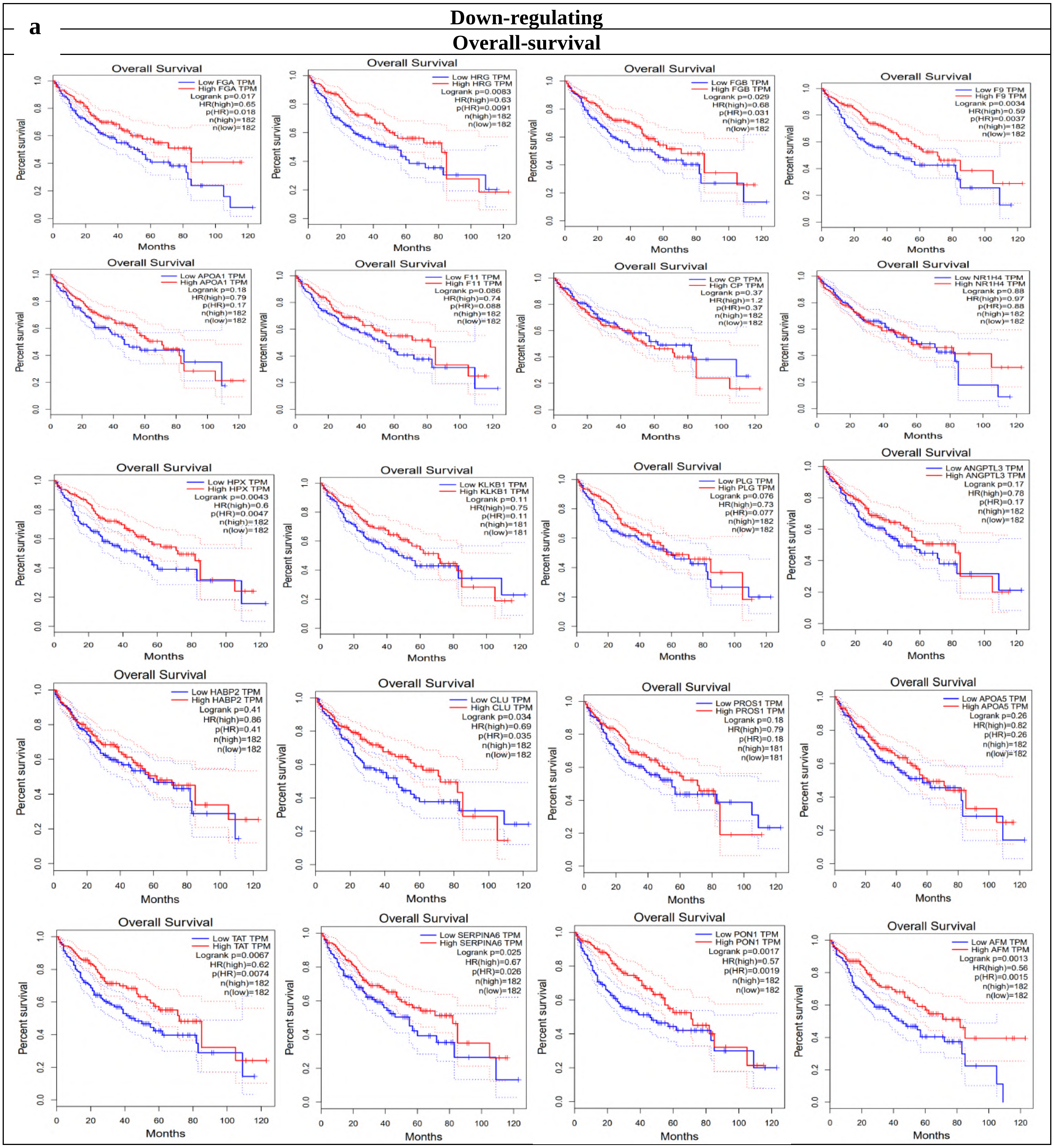
a
Down-regulating
Overall-survival
Overall Survival
Low FGA TPM
High FGA TPM
Logrank p=0.017
HR(high)=0.65
p(HR)=0.018
n(high)=182
n(low)=182
Overall Survival
Low HRG TPM
High HRG TPM
Logrank p=0.0083
HR(high)=0.63
p(HR)=0.0091
n(high)=182
n(low)=182
Overall Survival
Low FGB TPM
High FGB TPM
Logrank p=0.029
HR(high)=0.68
p(HR)=0.031
n(high)=182
n(low)=182
Overall Survival
Low F9 TPM
High F9 TPM
Logrank p=0.0034
HR(high)=0.59
p(HR)=0.0037
n(high)=182
n(low)=182
Overall Survival
Low APOA1 TPM
High APOA1 TPM
Logrank p=0.18
HR(high)=0.79
p(HR)=0.17
n(high)=182
n(low)=182
Overall Survival
Low F11 TPM
High F11 TPM
Logrank p=0.086
HR(high)=0.74
p(HR)=0.088
n(high)=182
n(low)=182
Overall Survival
Low CP TPM
High CP TPM
Logrank p=0.37
HR(high)=1.2
p(HR)=0.37
n(high)=182
n(low)=182
Overall Survival
Low NR1H4 TPM
High NR1H4 TPM
Logrank p=0.88
HR(high)=0.97
p(HR)=0.88
n(high)=182
n(low)=182
Overall Survival
Low HPX TPM
High HPX TPM
Logrank p=0.0043
HR(high)=0.6
p(HR)=0.0047
n(high)=182
n(low)=182
Overall Survival
Low KLKB1 TPM
High KLKB1 TPM
Logrank p=0.11
HR(high)=0.75
p(HR)=0.11
n(high)=181
n(low)=181
Overall Survival
Low PLG TPM
High PLG TPM
Logrank p=0.076
HR(high)=0.73
p(HR)=0.077
n(high)=182
n(low)=182
Overall Survival
Low ANGPTL3 TPM
High ANGPTL3 TPM
Logrank p=0.17
HR(high)=0.78
p(HR)=0.17
n(high)=182
n(low)=182
Overall Survival
Low HABP2 TPM
High HABP2 TPM
Logrank p=0.41
HR(high)=0.86
p(HR)=0.41
n(high)=182
n(low)=182
Overall Survival
Low CLU TPM
High CLU TPM
Logrank p=0.034
HR(high)=0.69
p(HR)=0.035
n(high)=182
n(low)=182
Overall Survival
Low PROS1 TPM
High PROS1 TPM
Logrank p=0.18
HR(high)=0.79
p(HR)=0.18
n(high)=181
n(low)=181
Overall Survival
Low APOA5 TPM
High APOA5 TPM
Logrank p=0.26
HR(high)=0.82
p(HR)=0.26
n(high)=182
n(low)=182
Overall Survival
Low TAT TPM
High TAT TPM
Logrank p=0.0067
HR(high)=0.62
p(HR)=0.0074
n(high)=182
n(low)=182
Overall Survival
Low SERPINA6 TPM
High SERPINA6 TPM
Logrank p=0.025
HR(high)=0.67
p(HR)=0.026
n(high)=182
n(low)=182
Overall Survival
Low PON1 TPM
High PON1 TPM
Logrank p=0.0017
HR(high)=0.57
p(HR)=0.0019
n(high)=182
n(low)=182
Overall Survival
Low AFM TPM
High AFM TPM
Logrank p=0.0013
HR(high)=0.56
p(HR)=0.0015
n(high)=182
n(low)=182
Percent survival
Months

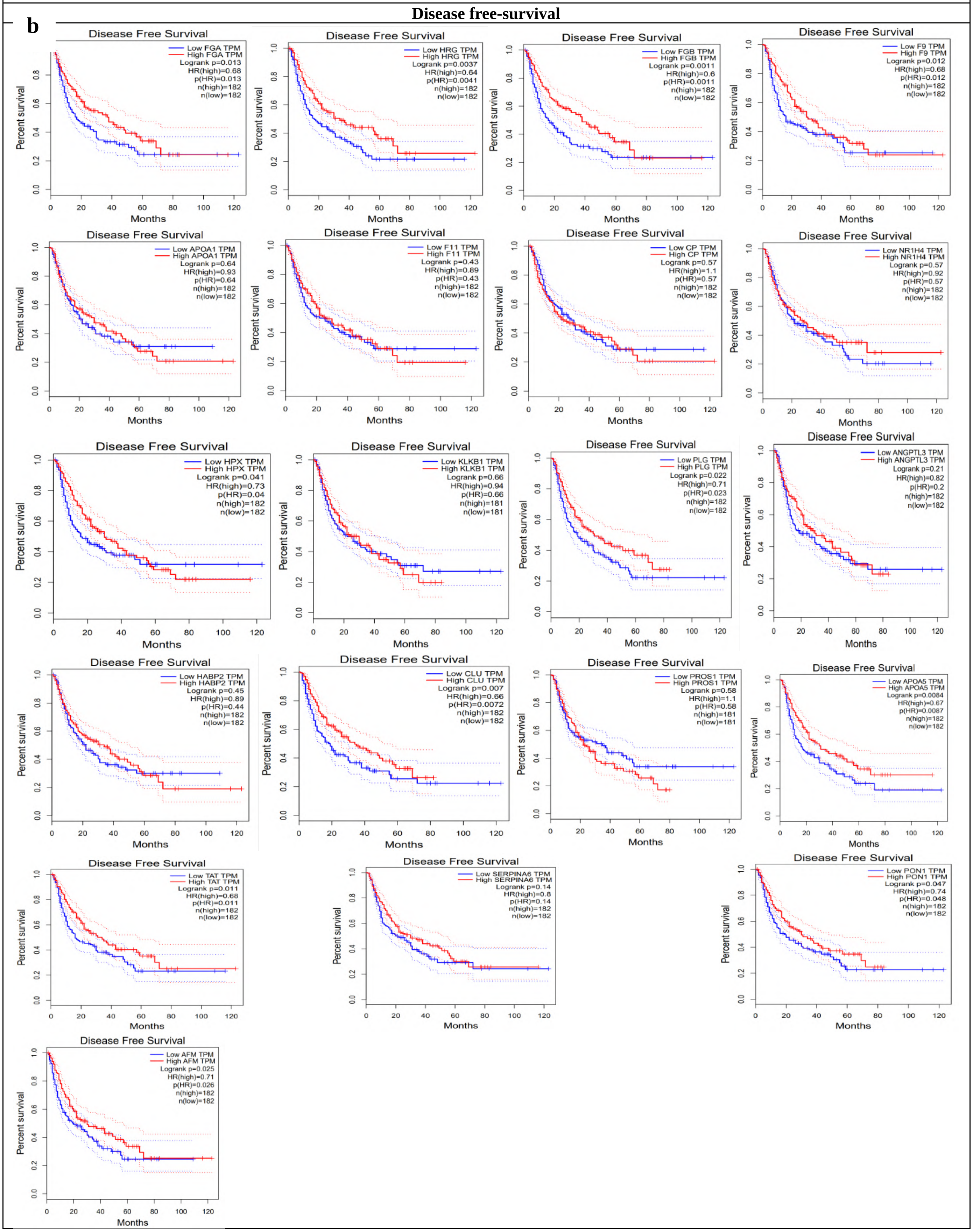

Fig. (S3. a, b). Overall Survival and Disease-free survival of downregulating genes obtained from Kaplan Meier plot using GEPIA.

Time is plotted on the X-axis, and survival chance is on the Y-axis. Genes that are downregulated and have changed in expression are shown by a red line, whereas a blue line shows genes that have not changed.

a

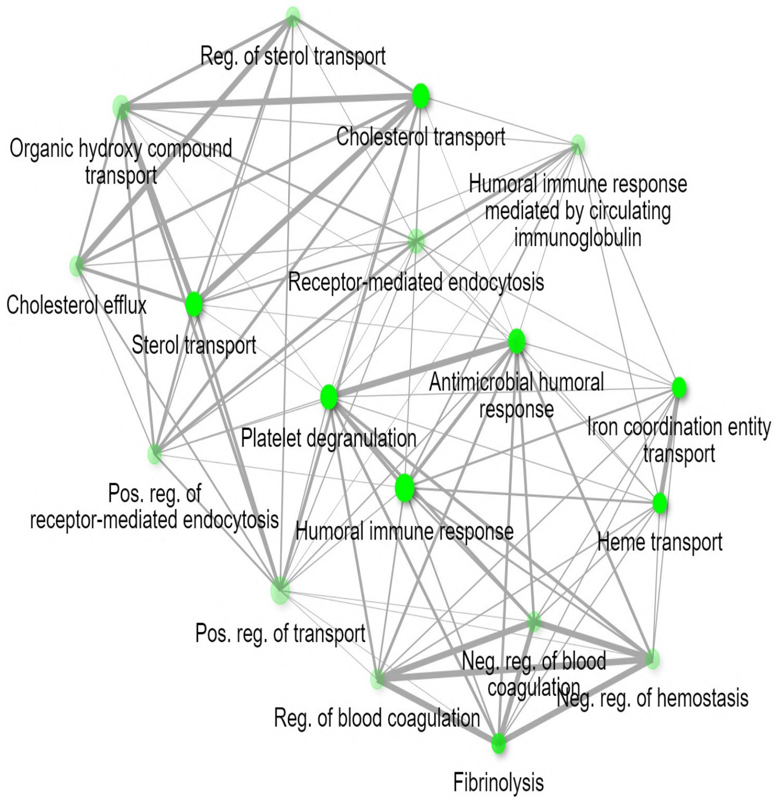

b

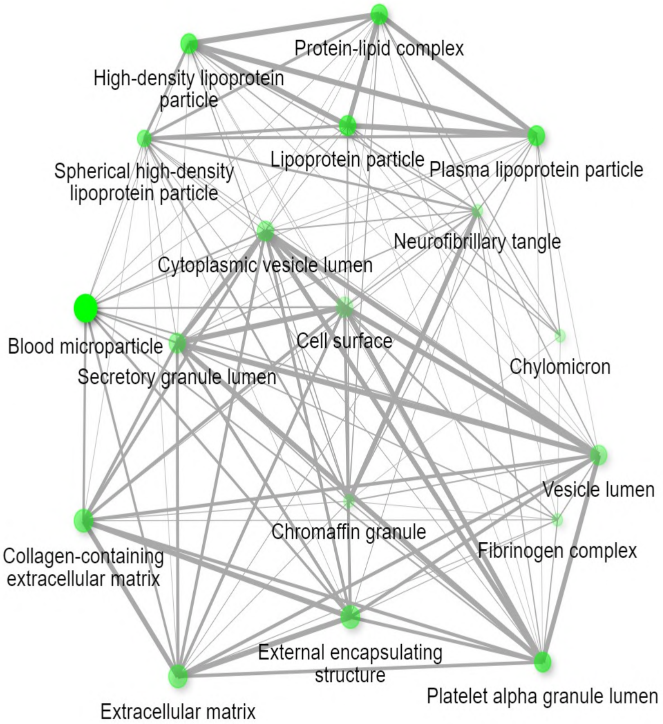

c

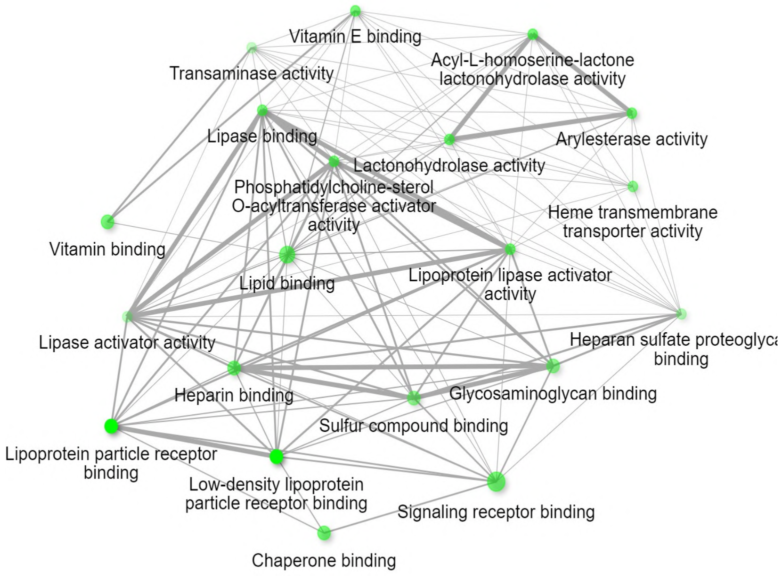

d

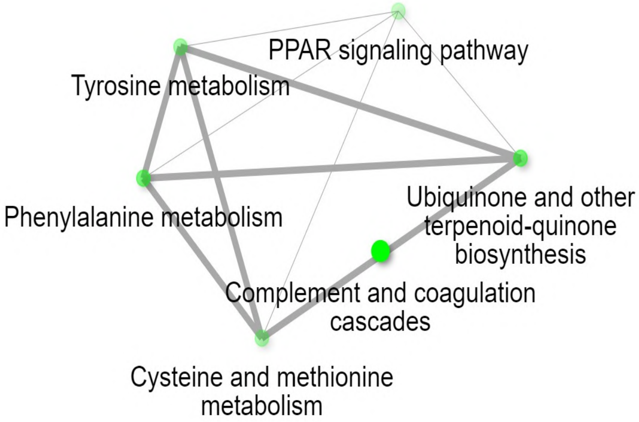

Fig. (S4). Function enrichment analysis and Gene Ontology Studies for downregulating genes, representing (**a**) Interconnection of biological processes, (**b**) Interconnection of cellular components, (**c**) Interconnection of molecular functions, and (**d**) KEGG Pathway Annotation. The bright, fluorescent green circle represents activities highly expressed by the genes.
